# Stimulation of CRISPR-mediated homology-directed repair by an engineered RAD18 variant

**DOI:** 10.1038/s41467-019-11105-z

**Published:** 2019-07-30

**Authors:** Tarun S. Nambiar, Pierre Billon, Giacomo Diedenhofen, Samuel B. Hayward, Angelo Taglialatela, Kunheng Cai, Jen-Wei Huang, Giuseppe Leuzzi, Raquel Cuella-Martin, Andrew Palacios, Anuj Gupta, Dieter Egli, Alberto Ciccia

**Affiliations:** 1 Department of Genetics and Development, Herbert Irving Comprehensive Cancer Center, Columbia University Irving Medical Center, New York, NY 10032 USA; 2 Naomi Berrie Diabetes Center and Department of Pediatrics, Columbia University Irving Medical Center, New York, NY 10032 USA; 3 Department of Experimental Medicine and Surgery, University of Rome Tor Vergata, Rome, 00133 Italy

**Keywords:** Genetic engineering, CRISPR-Cas9 genome editing, DNA recombination

## Abstract

Precise editing of genomic DNA can be achieved upon repair of CRISPR-induced DNA double-stranded breaks (DSBs) by homology-directed repair (HDR). However, the efficiency of this process is limited by DSB repair pathways competing with HDR, such as non-homologous end joining (NHEJ). Here we individually express in human cells 204 open reading frames involved in the DNA damage response (DDR) and determine their impact on CRISPR-mediated HDR. From these studies, we identify RAD18 as a stimulator of CRISPR-mediated HDR. By defining the RAD18 domains required to promote HDR, we derive an enhanced RAD18 variant (e18) that stimulates CRISPR-mediated HDR in multiple human cell types, including embryonic stem cells. Mechanistically, e18 induces HDR by suppressing the localization of the NHEJ-promoting factor 53BP1 to DSBs. Altogether, this study identifies e18 as an enhancer of CRISPR-mediated HDR and highlights the promise of engineering DDR factors to augment the efficiency of precision genome editing.

## Abstract (web summary)

Manipulating DNA repair pathways can be used to improve the outcomes of CRISPR-based genome editing. Here the authors derive an enhanced RAD18 variant that suppresses 53BP1 recruitment to DNA double-strand breaks to enhance homology-mediated repair.

## Introduction

Genome editing using CRISPR technology relies on the repair of site-specific DNA double-strand breaks (DSBs) induced by the RNA-guided Cas9 endonuclease^1^. Homology-directed repair (HDR) of these DSBs enables precise editing of the genome by introducing defined genomic changes, including base substitutions, sequence insertions, and deletions^2^. In genome editing experiments, HDR is stimulated by homologous donor templates delivered in the form of single-stranded oligodeoxynucleotides (ssODNs) or double-stranded DNA (dsDNA) donors^3,4^. However, the efficiency of HDR-dependent precision genome editing is limited by DSB repair pathways that compete with HDR, such as non-homologous end joining (NHEJ)^3^. The choice of DSB repair pathway is determined in large part by DSB resection, a nucleolytic process that converts DSB ends into 3′-single-stranded DNA overhangs^5^. Certain NHEJ factors, including 53BP1, promote the direct joining of DSBs by protecting DNA ends from resection^6–12^. Limited resection of DSB ends can expose regions of sequence microhomology, which favor DSB repair through microhomology-mediated end joining (MMEJ)^13,14^, while more extensive DSB resection generates the long 3′-single-stranded DNA tails required for HDR^3,15^. Thus, cellular factors that impede DSB resection represent major barriers to HDR-mediated precision genome editing.

Previous studies have shown that the efficiency of CRISPR-mediated HDR can be improved by modulating the cellular pathways responsible for DSB repair. For example, stimulation of HDR-promoting factors, such as the RAD51 recombinase^16,17^, or inhibition of NHEJ factors by small molecules or shRNA-mediated silencing have led to increased HDR efficiency^18–23^. In addition, Cas9-mediated HDR can be enhanced by the delivery of genetically-encoded HDR-promoting factors together with Cas9, single guide RNAs (sgRNAs) and DNA donor templates^17,18,22,24^. Such genetically-encoded elements have advantages over small molecule- and RNAi-based approaches, as they are amenable to genetic engineering via mutagenesis and can be delivered into cells in different formats (i.e., DNA, RNA or protein). These benefits have been demonstrated with i53, an engineered variant of ubiquitin that inhibits 53BP1 and enhances HDR when co-expressed with Cas9 in mammalian cells^22^. Similarly, ssODN-mediated HDR can be augmented by ectopic expression of a dominant-negative 53BP1 variant (dn53BP1) along with the DNA annealing factor RAD52^24^. In addition, improved transgene insertion at Cas9-induced DSBs was recently obtained by fusing the N-terminal domain of CtIP, an HDR-promoting factor that facilitates DSB resection, to Cas9^25^. Despite these important studies highlighting the influence of selected DNA repair factors on precision genome editing, the impact of proteins of the DNA damage response (DDR) on Cas9-induced HDR has not been systematically examined.

In this study, we conduct an unbiased screen of 204 human DDR open reading frames (ORFs) to evaluate their effect on CRISPR-mediated HDR and identify RAD18, a RING-type E3 ubiquitin ligase involved in post-replication repair and HDR^26–30^, as the most potent stimulator of both ssODN- and dsDNA-mediated HDR. Through functional analysis, we define the domains of RAD18 necessary to promote HDR at Cas9-induced DSBs and derive an enhanced RAD18 variant (e18) that stimulates HDR with greater efficiency and specificity. The e18 variant promotes HDR events by blocking the recruitment of 53BP1 to DSBs, and thereby inhibiting competition from the NHEJ pathway. In addition, we demonstrate that e18 augments Cas9-induced HDR at multiple genomic loci in various human cell types, including human embryonic stem cells. Altogether, this work establishes the engineered e18 variant of RAD18 as a potent enhancer of precision genome editing by CRISPR-dependent HDR.

## Results

### RAD18 is a potent enhancer of Cas9-mediated HDR

**Fig. 1:**
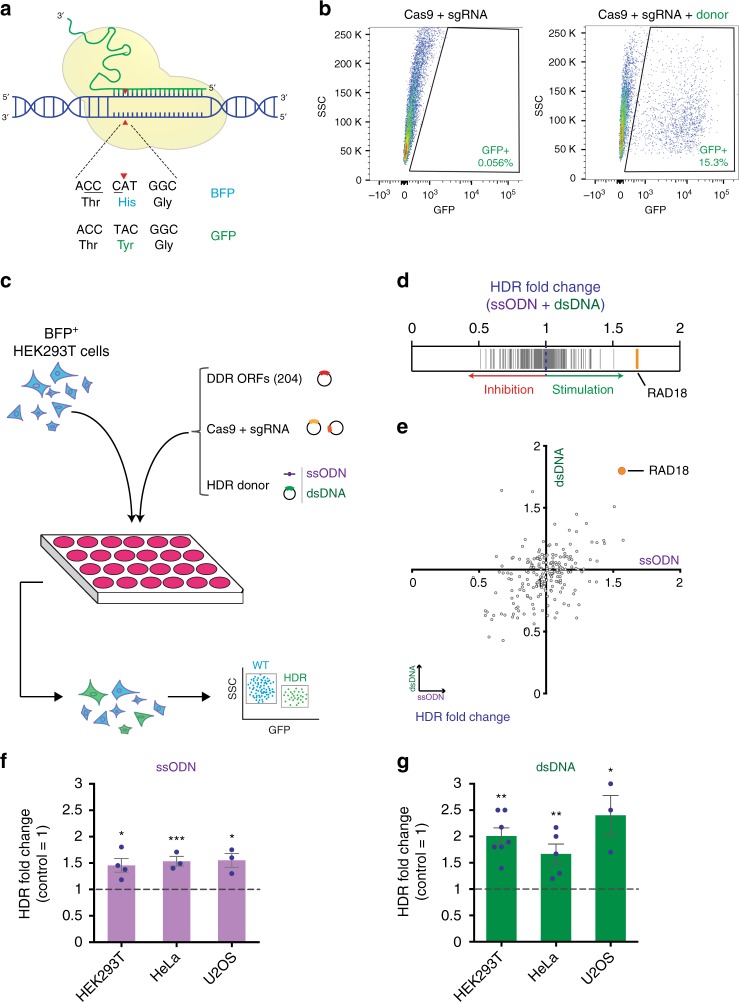
ORF screen to identify stimulators of CRISPR-mediated HDR. **a** Schematic of the BFP reporter utilized for the HDR assays. The sequences of the unedited (BFP) and edited (GFP) loci are shown. The PAM sequence is underlined and the site of Cas9-induced DNA cleavage is indicated by red arrows. **b** Representative flow cytometry plots of HEK293T cells carrying the BFP reporter shown in **a** targeted with Cas9 with or without transfection of an ssODN donor containing the GFP sequence indicated in **a**. **c** Experimental workflow for the arrayed ORF screen conducted in this study. HEK293T cells carrying the BFP reporter shown in **a** were seeded in 24-well plates and transfected with Cas9/sgRNA-expressing vectors and ssODN or dsDNA donors, in combination with individual human DDR ORFs (204). The percentage of GFP^+^ HDR events induced by the expression of each ORF was quantified by flow cytometry. **d** Graphical representation of the HDR levels for 204 DDR ORFs. HDR fold change corresponds to the average of the HDR values obtained using both ssODN and dsDNA donors for each ORF relative to control (HDR fold change = 1). Each line corresponds to a single ORF. The top HDR-stimulatory ORF (RAD18) is highlighted in orange. **e** Scatter plot of the HDR fold change (relative to control) induced by ssODN (x-axis) and dsDNA (y-axis) donors for 204 DDR ORFs. Each data point corresponds to the mean HDR fold stimulation of two biological replicates obtained for a single ORF using ssODN or dsDNA donors. RAD18 is highlighted in orange. **f**, **g** CRISPR-mediated HDR levels measured using the BFP reporter integrated in the indicated cell lines following transfection with ssODN (**f**) or dsDNA donor (**g**), and vectors expressing Cas9/sgRNA along with WT RAD18 or an empty vector control. In all experiments, cells were analyzed by flow cytometry for GFP fluorescence 72 h post transfection. The values of individual experiments were normalized to the empty vector control condition (dashed line) and presented as the mean ± s.e.m. (*n* ≥ 3). Statistical significance was calculated using a one-way analysis of variance with Tukey’s multiple comparison test, with a single pooled variance (*p < 0.05, **p < 0.01, ***p < 0.001). Source data are available in the Source Data file

To identify factors that stimulate precision genome editing by CRISPR-dependent HDR, we used a previously described fluorescence-based reporter that measures Cas9-induced HDR events^31^. This reporter consists of a stably integrated blue fluorescent protein (BFP) sequence that is converted into a green fluorescent protein (GFP) sequence following Cas9-induced cleavage and HDR-mediated substitution of a single BFP codon (CAT into TAC) (Fig. 1a). The percentage of cells that have undergone HDR-mediated repair of Cas9-induced DSBs can then be determined by monitoring the proportion of GFP-positive cells by flow cytometry (Fig. 1b). Using the above BFP reporter stably integrated in HEK293T cells, we examined the levels of HDR modulation induced by individually co-expressing, with Cas9-sgRNA, 204 ORFs implicated in DNA damage, repair and replication, as well as predicted interactors of key DNA repair proteins^32^ (Fig. 1c, Supplementary Data 1 and 4). In this assay, we monitored the frequency of HDR events at the BFP reporter using either ssODNs or dsDNAs as donor templates (Fig. 1c). Previous studies have shown that genome editing using dsDNA donors relies on canonical RAD51-mediated HDR, also known as homologous recombination, while ssODN-mediated genome editing depends on single-stranded template repair (SSTR), a RAD51-independent HDR process that is promoted by RAD52 and proteins of the Fanconi anemia pathway^4,24,33–36^. In agreement with the above findings^24^, expression of the RAD52 ORF elicited a 1.2–1.3-fold induction of ssODN-mediated HDR in HEK293T cells, thereby serving as a positive control in our assay (Supplementary Data 1). ORFs that modulated HDR ≥1.25- or ≤0.75-fold in the screen described above were individually validated in triplicates in HEK293T and HeLa cells harboring the BFP reporter (Supplementary Data 1). Our screen revealed that expression of the RAD18 ORF in HEK293T cells led to the greatest HDR-stimulatory effect for both ssODN and dsDNA donors (Fig. 1d, e). The HDR-stimulatory effect of RAD18 was also observed in HeLa and U2OS cells carrying the BFP reporter using both ssODNs and dsDNA donors (Fig. 1f, g). These studies identify RAD18 as a potent stimulator of CRISPR-mediated HDR events.

### RAD18 promotes Cas9-mediated HDR through its UBZ motif

**Fig. 2:**
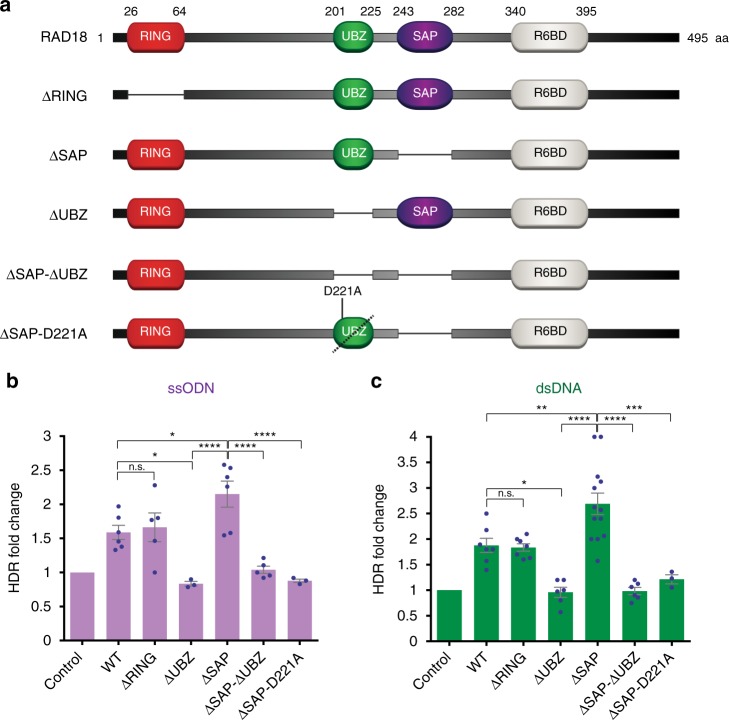
Analysis of RAD18 domains required to promote Cas9-mediated HDR. **a** Schematic diagram of wild-type RAD18 and its mutant variants generated in this study.  **b**, **c** HDR levels measured using the BFP reporter stably expressed in HEK293T cells following transfection of a BFP ssODN (**b**) or dsDNA donor (**c**), and vectors expressing Cas9 and BFP sgRNA, along with WT or mutant RAD18 variants, or an empty vector control. Experiments were conducted as described in Fig. 1f, g. The values of individual experiments were normalized to the empty vector condition and presented along with the mean ± s.e.m. (*n* ≥ 3). Statistical significance was calculated using a one-way analysis of variance with Tukey’s multiple comparison test, with a single pooled variance (*p < 0.05, **p < 0.01, ***p < 0.001, ****p < 0.0001). Source data are available in the Source Data file

RAD18 is an E3 ubiquitin ligase involved in post-replication repair (PRR), a cellular pathway that promotes the bypass of DNA lesions during DNA replication or after its completion^37^. PRR requires the ubiquitination of the DNA polymerase sliding clamp PCNA catalyzed by RAD18 in complex with the E2 ubiquitin ligase RAD6. Ubiquitination of PCNA by RAD18/RAD6 is dependent on the RING, SAP (SAF-A/B, Acinus and PIAS), and RAD6-binding domains of RAD18^29,38,39^. In addition, RAD18 also promotes DSB repair by homologous recombination^26–28,30^. Although its role in HDR is less well-characterized, both the UBZ and RING domains of RAD18 have been proposed to contribute to HDR^26,30^. To ascertain how RAD18 expression promotes CRISPR-mediated HDR, we constructed a panel of RAD18 mutants in which RAD18 functional domains have been individually removed (Fig. 2a). Upon expression of these RAD18 mutants in HEK293T cells carrying the BFP reporter (Supplementary Fig. 1a), we noted that HDR stimulation was unaffected by deletion of the RING domain, suggesting that the E3 ligase activity of RAD18 is dispensable for CRISPR-mediated HDR (Fig. 2b, c). In contrast, however, HDR was completely abrogated by deletion of the UBZ motif (Fig. 2b, c). Moreover, expression of the UBZ domain alone fused to an NLS sequence (UBZ-NLS) was sufficient to promote HDR (Supplementary Fig. 2), indicating that RAD18 stimulates CRISPR-induced HDR in a UBZ-dependent manner.

### e18 is an enhanced RAD18 variant for HDR stimulation

Interestingly, expression of the RAD18-ΔSAP mutant generated a significant increase in CRISPR-mediated HDR compared to full-length RAD18 or UBZ-NLS (Fig. 2b, c and Supplementary Fig. 2b). The enhanced effect of the ΔSAP mutant over full-length RAD18 was also confirmed using a distinct expression plasmid (Supplementary Fig. 1b, c). Of note, the HDR-stimulatory effect of RAD18-ΔSAP was fully abrogated upon deletion of the UBZ motif (Fig. 2b, c). To determine whether ubiquitin binding by the UBZ domain is required for HDR stimulation by RAD18-ΔSAP, we introduced the D221A mutation, which abrogates RAD18 ubiquitin binding^40,41^, into the ΔSAP mutant. As observed in Fig. 2b, c, the D221A mutation inhibited HDR stimulation by RAD18-ΔSAP in a manner comparable to deletion of the UBZ motif, indicating that ubiquitin binding is necessary for stimulation of CRISPR-mediated HDR by RAD18-ΔSAP. Similar results were observed upon electroporation of RAD18-ΔSAP mRNA into BFP^+^ HEK293T cells. As shown in Supplementary Fig. 1d, RAD18-ΔSAP mRNA increased Cas9-mediated HDR in a dose-dependent manner up to 3.9-fold over RAD18-ΔSAP-D221A mRNA, further confirming the role of UBZ-dependent ubiquitin binding in stimulating CRISPR-mediated HDR. These studies identify RAD18-ΔSAP as the minimal RAD18 construct that most efficiently stimulates CRISPR-dependent HDR and demonstrate that HDR stimulation by RAD18-ΔSAP can be achieved by mRNA delivery.

Previous studies have shown that RAD18 can induce PCNA ubiquitination on lysine 164 (K164) and promote the association between ubiquitinated PCNA and PRR factors^42^. To determine whether the HDR-stimulatory effect of RAD18 is dependent on its ability to ubiquitinate PCNA, we monitored the levels of PCNA ubiquitination on K164 in cells expressing the WT or ΔSAP-mutant forms of RAD18 with or without replication stress induced by UV radiation. Under both untreated and UV-treated conditions, K164 ubiquitination of PCNA was markedly reduced in cells expressing the ΔSAP mutant relative to those expressing WT RAD18 (Supplementary Fig. 1e), in agreement with previous work implicating the SAP domain of RAD18 in PCNA ubiquitination^39^. These observations confirm that stimulation of CRISPR-mediated HDR by RAD18 is independent of its role in inducing PCNA ubiquitination and establish that the ΔSAP mutant has enhanced specificity for HDR compared to WT RAD18. Given the higher HDR efficiency and specificity of the ΔSAP mutant relative to WT RAD18, we renamed the ΔSAP mutant as enhanced RAD18 (e18) variant for CRISPR-mediated HDR stimulation.

### e18 stimulates HDR by inhibiting 53BP1 localization to DSBs

**Fig. 3:**
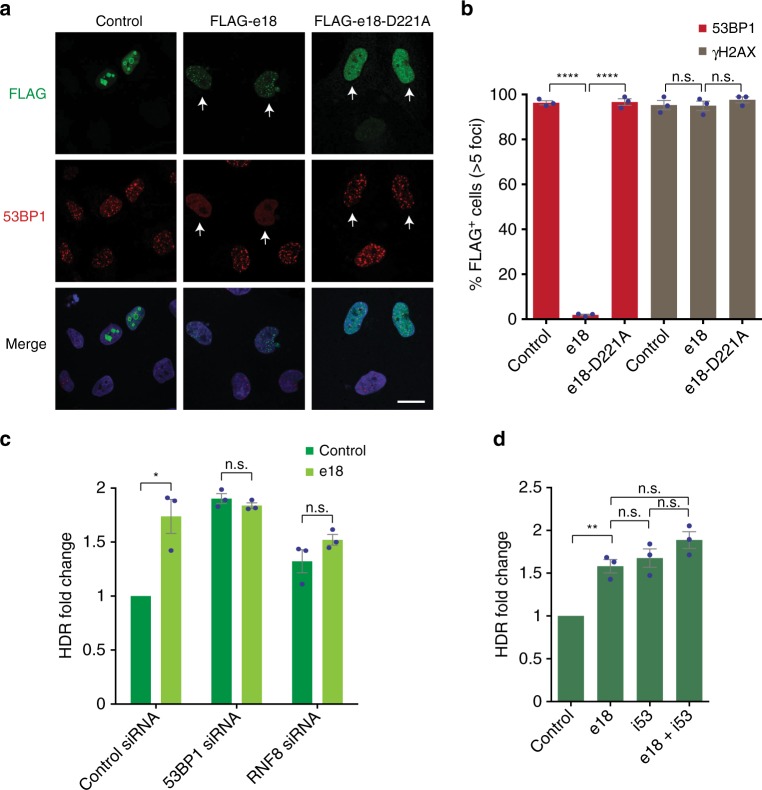
Interplay between e18 (RAD18-ΔSAP) and 53BP1 at DSBs. **a** Representative images showing IR-induced foci of the indicated proteins in U2OS cells transfected with vectors expressing FLAG-tagged e18, e18-D221A mutant or an empty vector control. Cells were fixed 1 h after irradiation with a 5 Gy dose and stained with antibodies that recognize the FLAG tag (green) or 53BP1 (red). Merged images with DAPI staining (blue) are also shown. Arrows indicate cells expressing e18 or e18-D221A. Scale bar: 16 μm. **b** Graphical representation of the percentage of FLAG-positive cells with >5 53BP1 or γH2AX foci, under the conditions described in **a**. Individual experimental data are shown along with the mean ± s.e.m. (*n* = 3). **c** HDR values in HEK293T cells carrying the BFP reporter and transfected with control, 53BP1 or RNF8 siRNAs, with or without e18 expression. In all experiments, 24 h after siRNA treatment, cells were transfected with Cas9 and BFP sgRNA expression vectors, a BFP dsDNA donor, and either an empty or e18-expressing plasmid. The percentage of GFP^+^ cells was then determined after 72 h by flow cytometry. The values of three individual experiments were normalized to the siRNA control condition and presented along with the mean ± s.e.m. (*n* = 3). **d** HDR levels in HEK293T cells carrying the BFP reporter transfected with Cas9 and BFP sgRNA expression plasmids and a BFP dsDNA donor, in the presence of e18, i53, or both. HDR frequency was determined by quantifying the percentage of GFP^+^ cells, as described in **c**. The values of individual experiments were normalized to the control and presented along with the mean ± s.e.m. (*n* = 3). Statistical significance of the data shown in **b**–**d** was calculated using a one-way analysis of variance with Tukey’s multiple comparison test, with a single pooled variance (*p < 0.05, **p < 0.01, ****p < 0.0001). Source data are available in the Source Data file

The UBZ domain of RAD18 binds the histone H2A ubiquitinated on lysine 15 (H2AK15ub), and this interaction can be abrogated by the D221A amino acid substitution^41^. Interestingly, H2AK15ub can also associate with the UDR domain of 53BP1 and is required for 53BP1 recruitment to DSBs^43^. Moreover, since RAD18 binds H2AK15ub with greater affinity than 53BP1, it can potentially displace 53BP1 from DSBs^30,41^. In agreement with these findings, we noted that e18, but not the e18-D221A mutant, localized to ionizing radiation-induced foci (IRIF) in U2OS cells and suppressed 53BP1 IRIF formation (Fig. 3a, b). In contrast, e18 expression did not alter IRIF formation by γH2AX, a marker of DSBs (Fig. 3b and Supplementary Fig. 3a). These findings indicate that e18 abrogates the recruitment of 53BP1 to DSBs in a manner dependent on H2AK15ub binding.

To determine whether e18-induced HDR stimulation was dependent on 53BP1, we depleted 53BP1 in HEK293T cells by siRNA and measured CRISPR-mediated HDR at the BFP reporter using dsDNA donors, as described above (Supplementary Fig. 3b). As previously observed^22^, 53BP1 depletion enhanced CRISPR-mediated HDR approximately 2-fold, in a manner comparable to e18 expression (Fig. 3c). Interestingly, e18 expression did not further stimulate HDR in 53BP1-depleted cells, suggesting an epistatic relationship between e18 expression and 53BP1 loss with respect to HDR stimulation (Fig. 3c). These observations were confirmed in e18-expressing cells using i53, a genetically-encoded inhibitor of 53BP1^22^ (Fig. 3d). In line with previous studies showing that the recruitment of both 53BP1 and RAD18 to DSBs is mediated by the RNF8-dependent ubiquitination pathway^26,44–46^, e18-mediated stimulation of HDR was not observed in cells depleted of RNF8 (Fig. 3c and Supplementary Fig. 3b). Taken together, these findings indicate that e18-dependent stimulation of CRISPR-mediated HDR occurs through the RNF8/53BP1 pathway.

### e18 expression leads to the inhibition of NHEJ

**Fig. 4:**
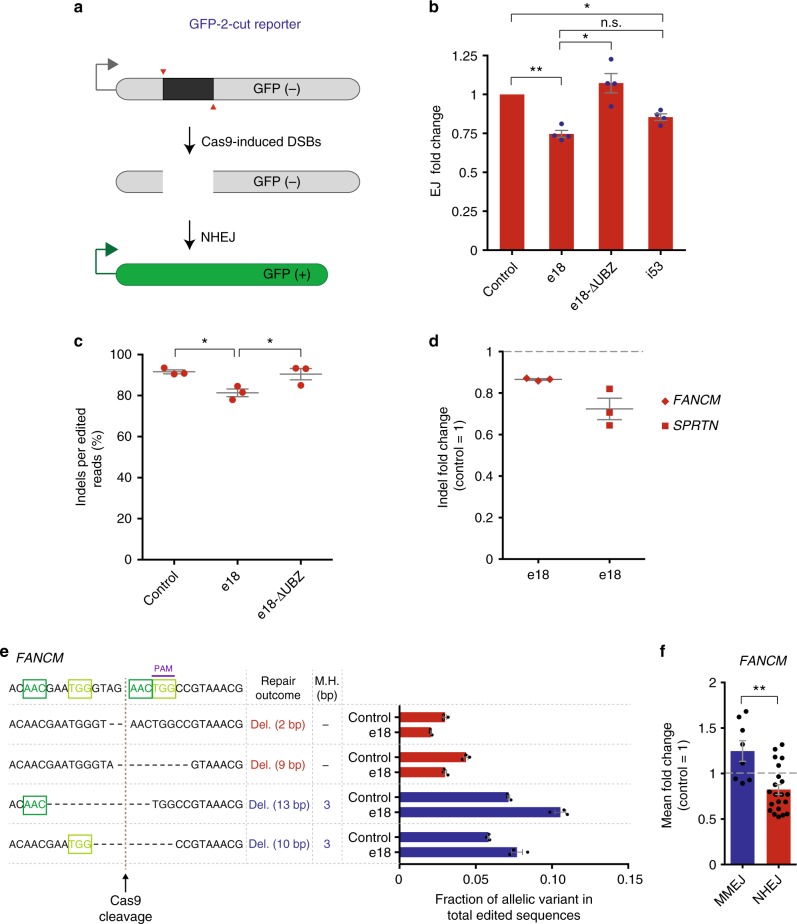
Indel levels at Cas9-induced DSBs after e18 expression. **a** Schematic of the GFP-2-cut reporter assay utilized to measure precise end joining. **b** Precise end joining (EJ) levels measured by flow cytometry using the GFP-2-cut reporter shown in **a** stably integrated in HEK293T cells transfected with vectors expressing Cas9/sgRNA along with empty vector control, e18, e18-ΔUBZ or i53. Individual experiments were normalized to the empty vector condition and presented along with the mean ± s.e.m. (*n* = 4). Statistical significance was calculated using a one-way analysis of variance with Tukey’s multiple comparison test, with a single pooled variance (*p < 0.05, **p < 0.01). **c** Indel levels measured by NGS at the BFP reporter integrated in HEK293T cells transfected with vectors expressing Cas9/sgRNA and dsDNA donor, along with e18, e18-ΔUBZ mutant or an empty vector control. Values are shown as percentage of indels per edited sequences (Indels + HDR). Individual data are presented along with the mean ± s.e.m. (*n* = 3). Statistical significance was calculated as in **b** (*p < 0.05). **d** Fold change in indel levels measured by NGS at the indicated loci in HEK293T cells transfected with vectors expressing Cas9/sgRNA along with e18 or an empty vector control. The indel values of individual experiments were normalized to the empty vector condition (dashed line) and presented as the mean ± s.e.m. (*n* = 3). **e** Pattern of indels induced upon treatment of HEK293T cells with Cas9 and sgRNA targeting *FANCM*, along with either an empty vector control or e18, as determined by NGS. The wild-type *FANCM* sequence is shown on the top together with 4 of the most frequent mutant alleles resulting from the repair of a Cas9-induced DSB (brown dashed line) in the *FANCM* locus. Sequences of microhomology (M.H., green boxes) and PAM sequence (purple line) are indicated. The repair outcome (deletion size) and the length of M.H. utilized for repair are specified. The frequency of the indicated repair products obtained upon transfection of an empty vector control or e18 is represented as fraction of total edited sequences along with the mean ± s.e.m. (*n* = 3). Non M.H.-dependent deletions and M.H.-mediated deletions are shown in red and blue, respectively. **f** Fold change in the mean of all MMEJ- and NHEJ-induced repair events upon targeting the *FANCM* locus shown in **e** with Cas9 in cells treated with e18, relative to empty vector control. Each data point represents the average, across 3 e18-treated biological replicates, of the fraction of each variant generated by MMEJ (blue) or NHEJ (red), as shown in **e**, relative to the empty vector control condition (dashed line). Data are presented as the mean ± s.e.m. (*n* = 3). Statistical significance was calculated with a paired *t*-test (**p < 0.01). See also Supplementary Data 2. Source data are available in the Source Data file

Previous studies have shown that inhibition of 53BP1 results in a reduction of NHEJ activity^47^. To determine the impact of e18 expression on NHEJ, we generated a reporter that measures precise end joining of Cas9-induced DSBs. Similar to a recently published reporter of precise end joining^48^, our reporter consists of a GFP gene inactivated by an intervening cassette, which, when removed upon Cas9-dependent cleavage at two distinct sites, leads to functional GFP detectable by flow cytometry (Fig. 4a and Supplementary Fig. 4a). Using this reporter, which we named GFP-2-cut, e18 expression reduced the efficiency of precise end joining in a manner comparable to i53 expression (Fig. 4b). This effect was dependent on the UBZ domain of e18, as deletion of this domain restored precise end joining to levels comparable to the empty vector control (Fig. 4b).

Repair of DSBs by NHEJ can result in the formation of small insertions and deletions (Indels). To investigate the effect of e18 expression on the frequency of indels generated by mutagenic end joining of DSBs, we analyzed by next-generation sequencing (NGS) the repair events occurring in e18-expressing cells at DSBs induced by Cas9 in the BFP reporter described above. In these studies, e18 expression led to a UBZ-dependent reduction in indel formation at the site of Cas9-mediated DNA cleavage (Fig. 4c). Reduction in indel formation was also observed at Cas9-induced DSBs in two distinct endogenous loci (*FANCM* and *SPRTN*), showing that e18 promotes CRISPR-mediated HDR at the expense of NHEJ (Fig. 4d and Supplementary Fig. 4b).

Limited resection of DSBs can favor MMEJ, a mutagenic repair pathway that competes with NHEJ to promote the joining of DSB ends containing regions of microhomology^13^. To determine whether MMEJ-dependent repair of Cas9-induced DSBs is altered in e18-expressing cells, we examined by NGS the pattern of end joining products generated at Cas9-induced DSBs in 2 endogenous loci (*FANCM* and *SPRTN*) upon e18 expression (Supplementary Data 2). As shown in Fig. 4e, f and Supplementary Fig. 4c–e, e18 expression promoted the formation of MMEJ-dependent repair products (microhomology-mediated deletions) at the expense of NHEJ-mediated repair products (insertions and deletions lacking microhomology), consistent with a possible role for e18 in stimulating the processing of DSB ends necessary for MMEJ. Overall, these findings further support the notion that e18 inhibits NHEJ by promoting DSB resection, which is required for both MMEJ and HDR pathways.

### e18 stimulates Cas9-mediated HDR at endogenous genomic loci

**Fig. 5:**
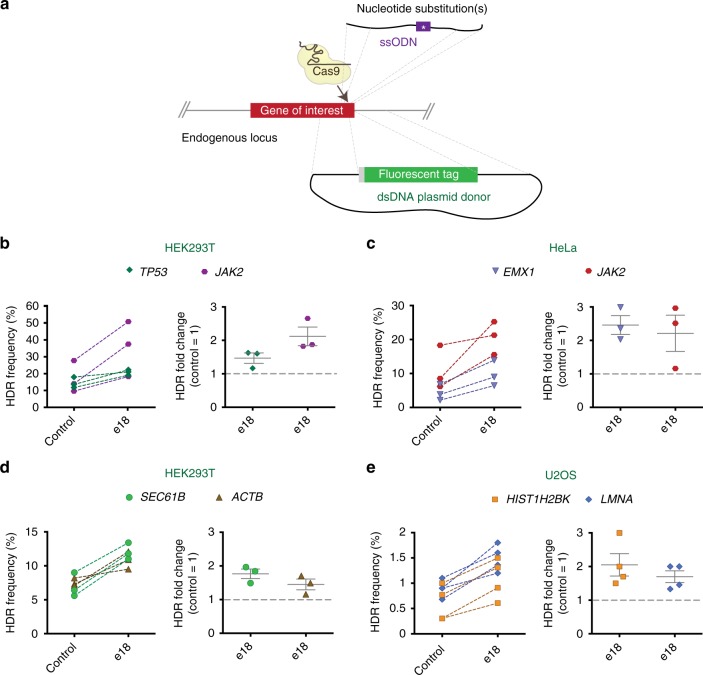
Targeting of endogenous loci in human cells using e18. **a** Schematics of the gene targeting experiments conducted using either ssODNs carrying nucleotide substitutions or dsDNA donor plasmids with fluorescent tags. **b**, **c** Gene targeting efficiency at the *TP53* (green) and *JAK2* (purple) loci in HEK293T cells (**b**) and the *EMX1* (blue) and *JAK2* (red) loci in HeLa cells (**c**) upon transfection of *TP53*, *JAK2*, or *EMX1* ssODN template, Cas9 and sgRNA expression vectors, and either a plasmid expressing e18 or an empty vector control. Gene targeting efficiency at the *TP53* locus was determined by NGS, while gene targeting efficiency at the *JAK2* and *EMX1* loci was estimated by RFLP assay. HDR frequency values of individual paired experimental sets are shown on the left graph. Fold change in HDR values for e18-expressing conditions relative to the control (dashed line) are shown on the right graph, where individual experiments are presented along with the mean ± s.e.m. (*n* = 3). **d** Gene targeting efficiency at the *SEC61B* (green) and *ACTB* (brown) loci in HEK293T cells transfected with either SEC61B-GFP or ACTB-GFP donor template, Cas9 and sgRNA expression vectors, and either a plasmid expressing e18 or an empty vector control, as determined by flow cytometry analysis of GFP^+^ cells. Experiments are represented as in **b** (n = 3). **e** Gene targeting efficiency at the *HIST1H2BK* (yellow) and *LMNA* (blue) loci in U2OS cells transfected with either HIST1H2BK-mAG or ACTB-Clover donor template, sgRNA and Cas9 expression vectors, and a plasmid expressing e18 or an empty vector control, as determined by flow cytometry analysis of mAG^+^ or Clover^+^ cells. Experiments are represented as in **b** (*n* = 4). The full list of HDR values for all the gene editing experiments at endogenous loci is available in Supplementary Data 3. Source data are available in the Source Data file

To test the efficiency of e18 in stimulating CRISPR-mediated HDR, we targeted several endogenous loci in multiple human cell lines using distinct DNA donor types (Fig. 5a and Supplementary Data 3). In particular, we investigated the effect of e18 on ssODN-mediated HDR by introducing a cancer-associated frameshift mutation into *TP53* (i.e., Arg209fs*6, 626_627delGA) and unique restriction sites into the *EMX1* and *JAK2* genes^24^. As shown in Fig. 5b, c and Supplementary Fig. 5a, e18 stimulated HDR using ssODN donors up to 2.7- and 3-fold in HEK293T and HeLa cells, respectively. Next, we examined the ability of e18 to enhance CRISPR-induced tagging of the *SEC61B* and *ACTB* genes with GFP using dsDNA plasmid donors^49^. In these assays, HDR events result in the expression of GFP-tagged proteins that can be detected by flow cytometry (Supplementary Fig. 5b). As shown in Fig. 5d, e18 enhanced HDR at the *ACTB* and *SEC61B* loci in HEK293T cells approximately 1.7- and 2-fold, respectively. We additionally examined whether e18 expression affected the efficiency of gene targeting at the histone H2B (*HIST1H2BK*) and *LMNA* loci in U2OS cells, using mAG and mClover dsDNA donor constructs, respectively^16,50^. These studies revealed that e18 expression enhanced gene targeting up to 3- and 2-fold at the *HIST1H2BK* and *LMNA* loci, respectively (Fig. 5e and Supplementary Fig. 5c). In agreement with our previous observations, expression of the e18-D221A mutant did not stimulate CRISPR-mediated HDR at endogenous loci, further confirming that the HDR-stimulatory effect of e18 is UBZ-dependent (Supplementary Data 3). Importantly, e18 expression did not alter cell cycle progression or cell proliferation, nor did it further sensitize cells to treatment with hydroxyurea, a replication stress-inducing agent, thereby indicating that e18 expression does not interfere with cellular replication (Supplementary Fig. 6).

**Fig. 6:**
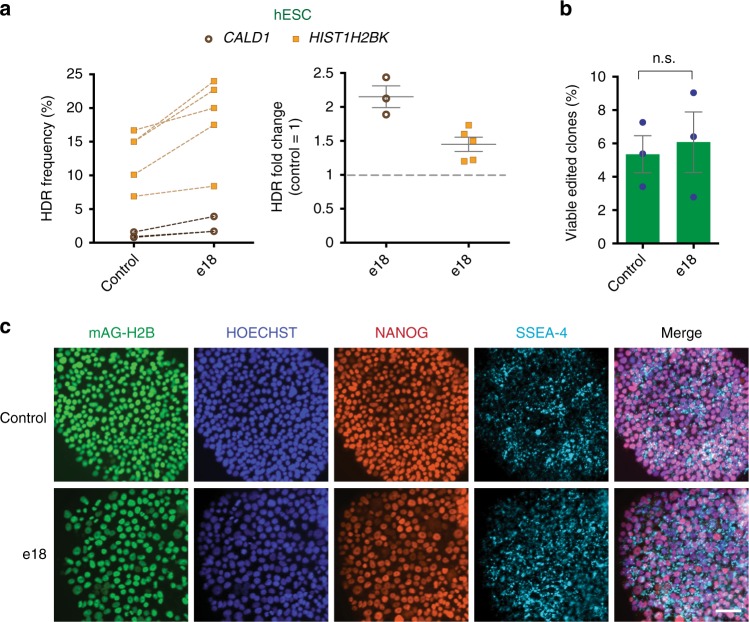
Targeting of endogenous loci in hESCs. **a** Gene targeting efficiency at the *CALD1* (brown) and *HIST1H2BK* (yellow) loci in hESCs pES12 transfected with a CALD1 ssODN or a HIST1H2BK-mAG plasmid donor, respectively, along with vectors expressing Cas9 and sgRNA, and either a plasmid expressing e18 or an empty vector control. Gene targeting efficiency at the *CALD1* and *HIST1H2BK* loci was determined by NGS and flow cytometry (mAG^+^ cells), respectively. Experiments were performed 5 days after transfection. HDR values are represented as in Fig. 5b (*HIST1H2BK*, *n* = 5; *CALD1*, *n* = 3). **b** Percentage of viable mAG^+^ hESC pES12 clones 14 days after transfection with Cas9/sgRNA-expressing vectors, mAG dsDNA donor targeting the *HIST1H2BK* locus, and either an empty or e18-expressing plasmid. The values of individual experiments are presented along with the mean ± s.e.m. (*n* = 3). Statistical significance was calculated using a paired *t*-test. **c** Immunostaining of pES12 cells edited at the *HIST1H2BK* locus using mAG dsDNA donors with or without e18 expression, as shown in **a**. Cell were stained using antibodies against the pluripotency markers NANOG (red) and SSEA-4 (cyan). mAG-tagged H2B is shown in green. Hoechst was used to detect DNA nuclei (blue). Merged images are also shown. Experiments were performed 5 days after transfection. Scale bar: 50 μm. Source data are available in the Source Data file

Human embryonic stem cells (hESCs) have emerged as an important tool for disease modeling, drug development, and tissue repair^51^. To demonstrate the utility of e18 as a stimulator of precision genome editing in hESCs, we introduced a point mutation in the *CALD1* gene using an ssODN^52^ and tagged the *HIST1H2BK* gene using an mAG dsDNA donor in the hESC line pES12. As shown in Fig. 6a, e18 enhanced CRISPR-mediated HDR at the *CALD1* and *HIST1H2BK* loci in hESCs up to 2.5- and 1.7-fold, respectively. Importantly, e18 did not affect the expression levels of the pluripotency markers NANOG and SSEA-4, nor the viability of hESCs (Fig. 6b, c and Supplementary Fig. 7b, c). These studies indicate that e18 can stimulate precision genome editing in hESCs without altering cellular viability and pluripotency.

## Discussion

Precision genome editing by the CRISPR-Cas9 system can be enhanced by modulating the cellular DNA repair machinery in a manner that promotes more efficient and precise repair of Cas9-induced DSBs. By screening 204 ORFs encoding various components of the DNA damage response, we have identified RAD18 as one of the most robust stimulators of CRISPR-mediated HDR (Fig. 1). Through functional studies, we derived an enhanced RAD18 variant, designated e18, with increased HDR-stimulatory activity and demonstrated the use of e18 as a genetically-encoded inhibitor of NHEJ that promotes Cas9-dependent precision genome editing in human cells using either ssODNs or dsDNA donor templates (Figs. 2–6).

The e18 variant of RAD18 lacks the SAP domain (RAD18-ΔSAP) (Fig. 2a), which is required for RAD18 binding to DNA replication fork structures and ubiquitination of PCNA during PRR^38,39^. Ubiquitinated PCNA is known to associate with translesion synthesis (TLS) DNA polymerases, which favor the bypass of DNA lesions in a potentially error-prone manner^37,53^. In accordance with these observations, PCNA ubiquitination was induced in human cells upon expression of WT RAD18, but not e18 (Supplementary Fig. 1e). Given its inability to catalyze PCNA ubiquitination, e18 is not expected to induce TLS-dependent DNA mutagenesis. In line with these conclusions, previous studies have shown that WT, but not ΔSAP-mutant, RAD18 is recruited to sites of UV-induced replication stress, where it promotes accumulation of the TLS polymerase POLH^39^. Since RAD18 variants that lack the SAP domain are defective in localizing to sites of replication damage^39^, e18 might be more readily available to associate with DSBs compared to WT RAD18, which localizes to both DSBs and stalled replication forks. The greater efficiency by which e18 stimulates HDR relative to WT RAD18 (Fig. 2b, c) might therefore result from its enhanced specificity for DSBs. Together, these observations indicate that e18 is a separation-of-function variant of RAD18 that is deficient for PRR but proficient for HDR. As such, e18 exhibits enhanced specificity for HDR relative to WT RAD18.

Our work indicates that e18 stimulates CRISPR-mediated HDR in a manner dependent on its UBZ motif (Fig. 2b, c and Supplementary Data 3). Previous studies reported that the UBZ motif of RAD18 binds to H2AK15ub with higher affinity than the UDR domain of 53BP1, suggesting that RAD18 can displace 53BP1 from chromatin near DSBs^30,41^. In line with these findings, we show that e18 abrogates the formation of 53BP1 IRIF through its UBZ motif (Fig. 3a, b). Furthermore, we show that e18-mediated 53BP1 displacement and stimulation of CRISPR-mediated HDR depends on D221, a key residue of the UBZ domain required for binding H2AK13/K15ub^40,41^ (Figs. 2b, c and 3a, b). Similar to RAD18, RNF169 has also been reported to bind H2AK13/K15ub through its MIU domain and inhibit 53BP1 IRIF formation^41,44,54^, raising the possibility that RNF169 expression might also stimulate CRISPR-mediated HDR through the displacement of 53BP1 from Cas9-induced DSBs. H2AK15 ubiquitination requires the RNF8 ubiquitin ligase, which promotes the UBZ-dependent recruitment of RAD18 to sites of DNA damage^26^. Since e18-mediated HDR stimulation is abrogated by RNF8 knockdown (Fig. 3c), the UBZ domain of e18 enhances HDR in an RNF8-dependent manner.

53BP1 has emerged as a promising target for increasing the efficiency of precision genome editing by HDR^22^. i53 enhances HDR at Cas9-induced DSBs by obstructing the Tudor domain of 53BP1^22^, and thereby inhibiting 53BP1 recruitment to chromatin^43^. Conversely, e18 functions by competitively binding to H2AK15ub, which prevents the UDR-dependent association of 53BP1 to chromatin in the vicinity of DSBs. Given that the Tudor, but not the UDR, domain of 53BP1 is required for non-DSB-dependent activities (e.g., transcriptional regulation) of 53BP1^55^, e18-mediated inhibition of 53BP1 recruitment to H2AK15ub might lead to fewer non-HDR-related effects compared to i53-dependent inhibition of 53BP1 Tudor domain. Our findings, therefore, provide an alternative means of inactivating 53BP1 to improve the efficiency of precision genome editing.

Inactivation of 53BP1 results in enhanced DSB resection, thus preventing NHEJ and favoring HDR^22,56^. In agreement with a role for e18 in inhibiting NHEJ, e18 reduces end joining and indel formation at Cas9-induced DSBs (Fig. 4). Consistent with the possible role of e18 in promoting DNA end processing, e18 expression stimulates MMEJ at the expense of NHEJ at Cas9-induced DSBs (Fig. 4e, f and Supplementary Fig. 4c–e). The above observations suggest that inhibition of MMEJ factors, such as POLQ^57^, might further stimulate Cas9-mediated HDR in e18-expressing cells. In line with this possibility, loss of POLQ was recently shown to increase the precision of gene targeting in NHEJ-deficient cells^58,59^. The possible role of e18 in stimulating DSB resection may also explain its ability to induce both ssODN- and dsDNA-mediated HDR. This feature distinguishes the use of e18 from other previously described HDR-stimulatory strategies involving expression of RAD52^24^ or treatment with the RAD51-enhancing small molecule RS-1^17^, which are specific for ssODN- or dsDNA-dependent HDR, respectively. Given that RAD51 and RAD52 promote invasion and annealing of resected DSB ends^15,60^, respectively, stimulation of their functions might synergize with e18 expression to further enhance CRISPR-mediated HDR. Additional stimulation of the HDR-promoting activity of e18 might be achieved by fusing DDR factors to e18 (or its UBZ motif), thereby facilitating their recruitment to DSBs through the binding of ubiquitinated H2A. As in the case of RAD18, other DDR factors could also be engineered to stimulate CRISPR-mediated HDR by eliminating domains that may attenuate their HDR-promoting activity. In this regard, some of the other hits identified in our screen (Supplementary Data 1), such as the BRCA1 partner BARD1, which recruits BRCA1 to DSBs to promote HDR^61–63^, would be attractive targets for engineered enhancement and either co-expression or fusion with e18. Collectively, our work highlights how rational engineering of DDR factors can enhance the efficiency of precision genome editing.

Our study demonstrates that co-delivery of e18 with Cas9 enables more efficient precision genome editing in multiple human cell lines (Figs. 5 and 6). Thus, the ability of e18 to robustly enhance ssODN-mediated HDR should also enable the production of precisely edited cell lines without the use of selectable markers, which can cause unintended perturbations of coding or non-coding genomic elements. Expression of e18 could facilitate marker-free genome editing when used in combination with HDR-enrichment approaches, such as the marker-free co-selection strategy based on modification of the *ATP1A1* gene^50^. Since e18 can promote the efficiency of gene tagging using dsDNA donor templates, it should also facilitate the study of the cellular localization and interactions of endogenously tagged proteins. Importantly, e18 stimulates CRISPR-mediated HDR in hESCs without altering cellular viability or pluripotency (Fig. 6 and Supplementary Fig. 7). This feature of e18 should facilitate the analysis of human genetic variants in distinct cell types derived by hESC differentiation. Given the growing number of genetic variants identified in the human population and in genetic diseases, these studies would be particularly informative to decipher cellular phenotypes caused by genetic variants. Altogether, our work establishes e18 as a robust tool for enhancing CRISPR-mediated precision genome editing in human cells, thereby aiding the study of gene function and modeling of human diseases.

## Methods

### DNA plasmids

Gateway pDONR223 or pDONR201 entry vectors containing DDR ORFs were either obtained from the Human ORFeome Library or individually cloned (Supplementary Data 4). Entry vectors were individually recombined into the pMSCV-N-HA-FLAG^64,65^ or pHAGE-HA^66^ vectors by Gateway cloning. The identity of inserts following recombination into destination vectors was confirmed by BsrGI digestion and/or Sanger sequencing. The RAD18 cDNA was cloned by Gibson assembly into pcDNA3.1(+) linearized with BamHI and EcoRI. RAD18 mutants were generated by site-directed mutagenesis of pDONR223-RAD18 with primers designed using NEBaseChanger (http://nebasechanger.neb.com). Mutants generated in the pDONR223 backbone were recombined into the pMSCV-N-HA-FLAG vector by Gateway cloning or inserted into pcDNA3.1(+) by Gibson assembly and validated by Sanger sequencing. The vectors pX330-U6-Chimeric_BB-CBh-hSpCas9 (a gift from Feng Zhang; Addgene plasmid #42230) and pU6-(BbsI)_CBh-Cas9-T2A-mCherry (a gift from Ralf Kuehn; Addgene plasmid # 64324) were utilized for Cas9 expression. All sgRNA sequences were cloned into B52^67^ (Addgene plasmid #100708) at BsmBI or BbsI restriction sites for sgRNA expression. The GFP-2-cut reporter was constructed by removing Cas9 from the lenti-SpCas9 blast plasmid (Addgene #104997) by XbaI and BamHI digestion. The plasmid was re-circularized using complementary oligonucleotides containing unique restriction sites. Oligonucleotide pairs were resuspended in TE (100 μM final concentration) and annealed in the following reaction buffer: 6.5 μl of water, 2 μl of 5X T4 ligase buffer (ThermoFisher Scientific), 0.5 μl of T4 PNK (NEB) and 0.5 μl of each oligonucleotide (100 μM; IDT). The reaction was conducted for 1 h at 37 °C, followed by incubation for 5 min at 95 °C and gradual temperature decrease from 95 to 15 °C. The annealed oligos were then ligated into the digested plasmid. The resulting plasmid, which was named B116 (Lenti-empty-Blast), was then digested with AgeI and BamHI (NEB) and the GFP-2-cut reporter cassette (ordered as a gBlock from IDT) was cloned using Gibson assembly. The GFP-2-cut reporter contains a 192 bp sequence inserted into the GFP sequence that was generated using a random DNA sequence generator (http://www.faculty.ucr.edu/~mmaduro/random.htm). STOP codons were removed manually from the randomly generated sequence and PAM motifs for Cas9 targeting were added at the junctions with the GFP sequence. A full list of the sequences of the sgRNAs, donor DNA templates and primers used for PCR amplification of genomic DNA loci or generation of RAD18 mutants can be found in Supplementary Data 4.

### Cell culture

U2OS, HeLa and HEK293T cells were obtained from American Type Culture Collection (ATCC). Cell lines were cultured in DMEM supplemented with 10% Fetalgro bovine growth serum (BGS, RMBIO) and 1X penicillin-streptomycin (ThermoFisher Scientific). Cells were grown at 37 °C with 5% CO_2_. To generate cell lines expressing the BFP reporter, HEK293T and HeLa cells were transduced with lentiviruses (multiplicity of infection [MOI] = 0.1) carrying the BFP reporter under the control of an EF1α promoter (Addgene #71825) and sorted by flow cytometry to produce a pure population of BFP-expressing cells. Single clones were generated from these populations and the clone exhibiting the highest HDR efficiency upon CRISPR-mediated targeting, as measured by BFP to GFP conversion, was selected for the ORF screen. Similar experiments were conducted in U2OS cells to produce a population of BFP-expressing cells. HEK293T cells with the GFP-2-cut reporter were generated by lentiviral infection (MOI = 0.1) and then selected with blasticidin. Diploid hESCs pES12^68^ were grown on 6-well plates treated with Geltrex Matrix (A1413302, ThermoFisher Scientific), with complete StemFlex medium (A3349401, ThermoFisher Scientific). Cells were grown at 37 °C in a humidified incubator with 5% CO_2_. The medium was replaced every day. Cell cultures were maintained 4–6 days until ~80% confluency and subcultured at a 1:6 to 1:10 dilution. Adherent cells were dissociated using TrypLE Express Enzyme (12605‐036, ThermoFisher Scientific). The medium was supplemented with 10 μM Rho-associated protein kinase (ROCK) inhibitor Y-27632 (S1049, Selleckchem) for one day after cell splitting in order to increase cell survival. Work with hESCs was reviewed and approved by the Columbia University ESCRO committee.

### Antibodies

The following primary antibodies were utilized for western blotting: anti-vinculin (V9131, Sigma-Aldrich; 1:5,000 dilution), anti-tubulin (NB600-506, Novus Biologicals, 1:5,000 dilution), anti-FLAG (A2220, Sigma-Aldrich; 1:5,000 dilution), anti-53BP1 (A300-273A, Bethyl Laboratories; 1:2,000 dilution), anti-RAD18 (9040, Cell Signaling Technology; 1:2,000 dilution), anti-RNF8 (sc-271462, Santa Cruz Biotechnology; 1:1,000 dilution), anti-PCNA (PC10, MA5-11358, ThermoFisher Scientific; 1:2,000 dilution), and anti-ubiquityl-PCNA (Lys164) (D5C7P, 13439, Cell Signaling Technologies; 1:10,000 dilution) antibody. The following antibodies were utilized for immunofluorescence studies: anti-53BP1 (A300-273A, Bethyl Laboratories; 1:1,000 dilution), anti-γH2AX (A303-837A, Bethyl Laboratories; 1:1,000 dilution), anti-FLAG (A2220, Sigma-Aldrich; 1:1,000 dilution), anti-human/mouse SSEA-4, mouse IgG (MAB1435, R&D Systems; 1:1,000 dilution), and anti-human/mouse NANOG, goat IgG (AF1997, R&D Systems; 1:1,000 dilution). We utilized the following secondary antibodies: HRP-conjugated AffiniPure goat anti-rabbit IgG (Jackson ImmunoResearch; 1:10,000 dilution), HRP-linked sheep anti-mouse IgG (NA931, GE Healthcare; 1:10,000 dilution), Alexa Fluor 488 goat anti-mouse and anti-rabbit IgG (ThermoFisher Scientific; 1:10,000 dilution), Alexa Fluor 647 goat anti-rabbit IgG (ThermoFisher Scientific; 1:5,000 dilution), Alexa Fluor 555 goat anti-mouse and anti-rabbit IgG (ThermoFisher Scientific; 1:5,000 dilution).

### Western blotting

Cell pellets were washed in PBS, resuspended in lysis buffer (100 mM Tris pH 6.8, 4% SDS and 1.716 M 2-mercaptoethanol) and incubated for 10 min at 95 °C. Proteins from the cell extracts were quantified using the Bradford Assay (Bio-Rad). Equal quantities of the extracts were diluted in 1X loading buffer (NuPage, ThermoFisher Scientific) and heated for 5 min at 95 °C. Samples were run on an 8% polyacrylamide gel at 160 V in tris-glycine buffer. Gels were subsequently transferred onto nitrocellulose membranes, which were then blocked for 30 min with 5% milk (Bio Basic) in TBS 0.1% Tween-20 (TBS-T). Membranes were then incubated with primary antibodies in TBS-T supplemented with 1% milk overnight at 4 °C. Membranes were then washed three times in TBS-T and incubated for 1 h with IgG secondary antibodies coupled with HRP at 1:1000 dilution in TBS-T/1% milk. Membranes were subsequently washed three more times in TBS-T and the HRP signal was detected using SuperSignal West Pico Chemiluminescent Substrate (ThermoFisher Scientific) and autoradiography films (Southern Labware).

### Immunofluorescence microscopy

U2OS cells were grown on glass coverslips, fixed and permeabilized simultaneously in 2% (w/v) paraformaldehyde, 0.25% (v/v) Triton X-100 in PBS for 30 min at room temperature. Cells were blocked with 3% BSA in PBS-T for 30 min at room temperature. Cells were then incubated with the primary antibody diluted in blocking buffer overnight at 4 °C. Cells were next washed with PBS and then incubated with secondary antibodies diluted in blocking buffer at room temperature. The coverslips were mounted onto glass slides with Flouroshield with DAPI (Sigma-Aldrich). Confocal images were taken using a Nikon A1 confocal microscope. Image collection, processing and analysis was performed in the Confocal and Specialized Microscopy Shared Resource of the Herbert Irving Comprehensive Cancer Center at Columbia University Irving Medical Center. pES12 cells edited at the *HIST1H2BK* locus with the mAG tag were sorted for mAG expression on a Bio-Rad S3e cell sorter. mAG-positive sorted cells were grown at low density in a 24-well plate until colonies formed, following which they were fixed with 4% PFA for 20 min at room temperature. The cells were then permeabilized with DPBS and 3% donkey serum with 0.1% Triton-X for 30 min at room temperature. Primary antibodies in blocking solution were then added to the cells and incubated overnight at 4 °C. Cells were then washed in PBST (0.1% Tween-20). Secondary antibodies and Hoechst in blocking solution were subsequently added to the cells and incubated for 1 h at room temperature. Cells were then washed with PBST and imaged using an Olympus IX-71.

### RNA interference

All siRNAs employed in this study were single-duplex siRNAs purchased from Horizon Dharmacon. RNA interference (RNAi) transfections were performed using HiPerFect Transfection Reagent (Qiagen) in a forward transfection mode. The individual siRNA duplexes used were targeting 53BP1/TP53BP1^22^ (D-003549-01, Horizon Dharmacon), control firefly luciferase (Horizon Dharmacon) and RNF8^69^ (Horizon Dharmacon). Except when stated otherwise, siRNAs were transfected 48 h before cell processing. The sequences of siRNAs used in this study can be found in Supplementary Data 4.

### ORF screen and HDR assays with the BFP reporter

The ORF screen was conducted as follows. BFP^+^ HEK293T cells were seeded at 50–70% confluency into 24-well plates and transfected by mixing polyethylenimine (PEI; 3 μl; 1 mg/ml solution; Sigma-Aldrich) and SpCas9 (250 ng), an sgRNA (250 ng) targeting BFP and a vector expressing a DDR ORF or an empty control plasmid (79 fmol), along with either a plasmid HDR donor (500 ng) or an ssODN (4 pmol). The cells were collected 3 days after transfection and analyzed by flow cytometry for GFP^+^ cells using a BD LSRFortessa. To overcome variations due to transfection efficiency and plate effect, multiple controls were used per plate and the percentage of GFP^+^ cells calculated for each sample was normalized to the mean of all the samples on a single plate. Biological duplicates were repeated on a separate day, with cells from different passages and the location of each DDR factor on the plate changed to avoid positional effects. Serial steps of validations were performed: first, a low cutoff of HDR modulation (<0.75- and >1.25-fold) was utilized for pre-selection of the top hits; second, the identified hits were taken forward and tested in biological triplicates in BFP^+^ HEK293T cells; third, the hits that continued to reproduce in these trials were then tested in three independent experiments in BFP^+^ HeLa cells. The effect of RAD18 WT and mutants, e18 and i53 on Cas9-induced HDR was examined in BFP^+^ HEK293T using the experimental conditions described above. The effect of WT RAD18 expression on HDR was also determined in BFP^+^ HeLa and U2OS cells, which were transfected with the above constructs and donor molecules using Transit-LT1 (3 μl; Mirus), under the conditions described for BFP^+^ HEK293T cells. To examine the levels of Cas9-induced HDR upon e18 expression and 53BP1 or RNF8 depletion, BFP^+^ HEK293T cells were transfected with control, 53BP1 or RNF8 siRNAs (10 nM) using HiPerFect Transfection Reagent (Qiagen). Twenty four hours later, the cells were transfected with Cas9 and sgRNA expression vectors, BFP dsDNA donor, and either an empty or e18-expressing pcDNA3.1 vector, under the above experimental conditions. Seventy two hours after plasmid transfection, the cells were trypsinized and the percentage of GFP^+^ cells was analyzed using the BD LSRFortessa flow cytometer.

### End joining assays with the GFP-2-cut reporter

Precise NHEJ was measured in HEK293T cells harboring the GFP-2-cut reporter generated as described above. In all experiments, 24-well plates were seeded at ~70% cell confluency and cells were transfected by mixing PEI (3 μl; 1 mg/ml solution) and plasmids expressing sgRNAs (250 ng) targeting the reporter and SpCas9 (250 ng), along with an empty vector or a plasmid expressing e18 or i53 (63 fmol). Precise NHEJ was quantified as the percentage of GFP^+^ cells measured by flow cytometry analysis.

### RFLP-based gene targeting assays

The RFLP assays were performed to detect HDR events at the *EMX1* and *JAK2* loci using our previously described method^67^, as detailed below. HEK293T and HeLa cells were transfected with plasmids expressing SpCas9 (250 ng) and EMX1 or JAK2 sgRNAs (250 ng), along with ssODNs (4 pmol) and either an empty or e18-expressing pcDNA3.1 vector (63 fmol). Transfection was conducted on HEK293T or HeLa cells seeded at 50–70% confluency into 24-well plates using PEI (3 μl; 1 mg/ml solution) or Transit-LT1 (3 μl), respectively. Three days after transfection, cells were harvested, the cell pellet was resuspended in the Quick Extract DNA Extraction Solution (Epicenter) and heated sequentially at 65 °C for 5 min and 95 °C for 5 min to isolate genomic DNA (gDNA). The isolated gDNA was quantified using Nanodrop, diluted in water and stored at −20 °C or directly used in PCR reactions. PCR reactions were performed using the isolated gDNA. The following oligonucleotides were utilized for this reaction: EMX1 (EMX1 PCR Forward and Reverse, Tm = 72 °C) and JAK2 (JAK2 PCR Forward and Reverse, Tm = 72 °C). The restriction digestion was performed with 2 μl of the PCR reaction mix supplemented with CutSmart Buffer (NEB) and one unit of PmeI. The restriction digestion was carried out for 1 h 30 min at 37 °C, following which the samples were loaded onto a 6% TBE polyacrylamide gel. Gel pictures were taken using LI-COR Odyssey. The band intensity was quantitated using ImageJ (v.1.51m9, http://imagej.nih.gov/ij). The percentage of HDR was calculated using the equation (*b* *+* *c*/*a* *+* *b* *+* *c*) × 100, in which *a* is the intensity of the uncleaved band and *b* and *c* are the intensities of the cleavage products.

### Fluorescence-based gene targeting assays

The *HIST1H2BK*, *LMNA, SEC61B* and *ACTB* targeting assays were performed in U2OS or HEK293T cells by transfection of the following plasmids: empty or e18-expressing pcDNA3.1 vector (63 fmol) and Cas9-P2A-mCherry (250 ng), along with an sgRNA vector (250 ng) and dsDNA donor (500 ng) specific for each targeted locus. Transfection was conducted on HEK293T or U2OS cells seeded at 50–70% confluency into 24-well plates using PEI (3 μl; 1 mg/ml solution) or Transit-LT1 (3 μl), respectively. Edited fluorescent cells were measured by flow cytometry on a BD LSRFortessa 3 days after transfection, and the results were analyzed using FlowJo v10 software. For editing in hESCs, cells were nucleofected at 80% confluency on a 6-well plate using the human embryonic stem cell Nucleofector Kit (VVPH-5012, Lonza) for the Nucleofector 2b device (AAB-1001, Lonza). One well was used per nucleofection reaction. Cells were dissociated with TrypLE express enzyme, centrifuged 4 min at 160*×g* and then resuspended in 100 μl of the nucleofection mix containing hESC nucleofection buffer with the following plasmids: empty or e18-expressing pcDNA3.1 vector (0.9 pmol), Cas9-P2A-mCherry (2.5 μg), HIST1H2BK sgRNA (1 μg), HIST1H2BK-mAG plasmid donor (5 μg). The complete reaction mix was added to the electroporation cuvette and underwent transfection with program A-23 of the Nucleofector 2b device. 48 h after nucleofection, cells were dissociated using TrypLE, filtered to obtain a single-cell solution, and subjected to FACS on the Bio-Rad S3e for mCherry-expressing cells. The number of sorted mCherry cells was kept consistent between conditions within each trial. During sorting cells were kept at 4 °C in StemFlex supplemented with ROCK inhibitor. mCherry-expressing cells were then reseeded and left to grow for 4–5 days, dissociated at a confluence of ~50% and analyzed for percentage of mAG^+^ cells on the BD LSRFortessa to determine HDR efficiency. The raw data of all HDR assays targeting endogenous loci in HEK293T, U2OS and HeLa cells and hESCs can be found in Supplementary Data 3. Flow cytometry analyses were conducted in the Herbert Irving Comprehensive Cancer Center Flow Cytometry Shared Resource.

### Next-generation sequencing-based HDR and end joining assays

NGS analysis was performed for genome editing experiments at the BFP reporter and at the *TP53*, *CALD1*, *FANCM*, and *SPRTN* loci. HEK293T cells (BFP, *TP53*, *FANCM*, and *SPRTN* loci) and pES12 cells (*CALD1* locus) were transfected or nucleofected, respectively, with a mixture of plasmids expressing SpCas9 and sgRNAs, and either an empty or e18-expressing pcDNA3.1 vector, under the experimental conditions described above. In the assays targeting BFP, *TP53* and *CALD1*, a dsDNA donor (BFP) or ssODN (*TP53*, *CALD1*) was also utilized. Five days after transfection/nucleofection, cells were collected and their gDNA was isolated as detailed above. Sequencing libraries were prepared using primers listed in Supplementary Data 4. The protocol outlined herein is modified from a protocol described elsewhere^68^. In the first PCR step, the targeted gene was amplified from 100 ng of gDNA in a 25 μl reaction with Q5 Master Mix (M0494L, NEB) and 500 nM final concentration of forward and reverse primers. This step was omitted for experiments in which the *FANCM* and *SPRTN* genes were targeted, as they did not include delivery of donor molecules. The thermal cycler program for the first PCR was as follows: 1 min at 98 °C, 35 cycles × 10 s at 98 °C, 20 s at 66 °C, 30 s at 72 °C, and 2 min at 72 °C. The second PCR was performed to add the Illumina P5 and P7 adaptors. The product from the first PCR was diluted 1:100 and 2 μl of this dilution was used as template. The thermal cycler program for the second PCR was as follows: 1 min at 98 °C, 35 cycles × 10 s at 98 °C, 20 s at 60 °C, 30 s at 72 °C, and 2 min at 72 °C. A third PCR was performed to add index barcodes to each sample. For this PCR, the product of the first PCR was diluted 1:100 and 8 μl of this dilution were used as template in a 25 μl reaction with Q5 Master Mix and 500 nM final concentration of each of the forward (i5) and reverse (i7) primers. The thermal cycler program for the third PCR was as follows: 1 min at 98 °C, 12 cycles × 10 s at 98 °C, 20 s at 60 °C, 30 s at 72 °C, and 2 min at 72 °C. PCR amplifications were verified using 2% agarose gels in TAE. The indexed amplicons from PCR #3 were pooled and gel purified. Gel purified samples were sequenced at the Genome Sciences Facility at The Pennsylvania State College of Medicine. Data was analyzed using CRISPResso^70^. The sequences and allelic frequencies of *FANCM* and *SPRTN* variants identified by NGS are available in Supplementary Data 2.

### HDR assays with mRNA delivery

Cas9 mRNA was obtained from TriLink (L-7206). sgRNA targeting the BFP reporter was generated by in vitro transcription using the HiScribe Quick T7 High Yield RNA Synthesis Kit (E2050, NEB) following manufacturer’s protocol. e18 and e18-D221A mRNAs were produced using the mMESSAGE mMACHINE T7 ULTRA Kit (ThermoFisher Scientific), according to the manufacturer's instructions. Cas9 mRNA (250 ng), BFP sgRNA (250 ng) and ssODN (5 fmol), along with e18 or e18-D221A mRNA (0–400 ng), were nucleofected into BFP^+^ HEK293T cells (100,000) using the Neon transfection system (ThermoFisher Scientific). The following electroporation parameters were used: 1,500 v (pulse voltage), 30 ms (pulse width), 1 (pulse number), 10 μl (tip). The cells were collected 3 days after nucleofection and analyzed by flow cytometry for GFP^+^ cells using a BD LSRFortessa. Oligonucleotide sequences used to produce the BFP sgRNA are available in Supplementary Data 4.

### Cell cycle analysis

HEK293T cells were plated at 50–70% confluency into 24-well plates and transfected with a pcDNA3.1 vector either empty or expressing e18 or the e18-D221A mutant (63 fmol). The cells were collected 2 days after transfection, centrifuged and washed in PBS, fixed in 70% ethanol and stored overnight at −20 °C. At the time of analysis, the cells were centrifuged, washed twice in PBS, resuspended in PBS with RNase (0.1 mg/ml) and propidium iodide 10 μg/ml, and incubated at 37 °C for 15–30 min in the dark. Cell cycle distribution was determined using the BD LSRFortessa machine and FlowJo v10 software.

### Cell proliferation and viability assays

HEK293T and U2OS cells were plated at 50–70% confluency into 24-well plates and transfected with a pcDNA3.1 vector either empty or expressing e18 or the e18-D221A mutant (63 fmol). After 24 h, the transfected cells were seeded at 20,000 cells/well and 10,000 cells/well, respectively, in a 96-well plate and monitored over 8 days. During the time course, cells were passaged into a larger plate upon reaching 70% confluency, to allow for continued proliferation. Cell proliferation was monitored across different time points by collecting the cells and quantifying them using the Countess II Automated Cell Counter (ThermoFisher Scientific) following the manufacturer’s protocol. For survival assays upon HU treatment, transfected U2OS cells were seeded in triplicate at 4000 cells/well in 12-well plates and treated after 16 h with DMSO or with the indicated concentrations of HU. 7 days after treatment, cells were fixed and stained using crystal violet solution (0.5% [w/v] in 20% methanol). For quantification, bound crystal violet was dissolved in 10% (v/v) acetic acid, and absorbance of 1:50 dilutions was measured at 595 nm using the SpectraMax iD3 microplate reader (Molecular Devices). For cell viability studies in hESCs, diploid pES12 cells were electroporated at 80% confluency in a six-well plate with plasmids expressing Cas9 (2.5 μg) and HIST1H2BK sgRNA (1 μg), and either an empty or e18-expressing pcDNA3.1 vector (0.9 pmol). As a positive control for apoptosis, cells were treated with cisplatin (50 μM) 24 h after plating. 5 days after nucleofection, apoptosis was assayed using the Annexin V-FITC Apoptosis Staining kit (Abcam) by flow cytometry using BD LSRFortessa. pES12 cells edited by HDR using a HIST1H2BK-mAG plasmid donor (5 μg; mAG-positive cells), following treatment with empty vector as control or e18, were sorted as single clones into each well of a 96-well plate using the Influx cell sorter machine. The viability of the clones was monitored over 14 days and the number of viable clones on day 14 was evaluated for each treatment condition.

### UV irradiation

HEK293T cells were transfected with pcDNA3.1 vectors either empty or expressing WT RAD18 or e18, as described above. Two days after transfection cells were exposed to 40 or 60 J/m^2^ radiation using a Stratalinker 2400. Cells were harvested 4 h after UV radiation.

### Reporting summary

Further information on research design is available in the Nature Research Reporting Summary linked to this article.

## Supplementary information

Supplementary Information

Supplementary Data 1

Supplementary Data 2

Supplementary Data 3

Supplementary Data 4

Reporting Summary

Description of Additional Supplementary Files

Source Data

## Data Availability

Sequencing data have been deposited into the NCBI database and are accessible as BioProject # PRJNA54470 https://www.ncbi.nlm.nih.gov/bioproject/PRJNA544700. The source data underlying Figs. 1d–g, 2b, c, 3b–d, 4b–f, 5b–e, 6a, b and Supplementary Figs. 1a–e, 2b, c, 3b, 4b–e, 5a, 6a–c, and 7a, c are provided as a Source Data file. All unique materials described in this manuscript will be made available upon request.
