# Supplementary material for: Stimulation of CRISPR-mediated homology-directed repair by an engineered RAD18 variant
Source: Nat Commun. 2019 Jul 30;10:3395. doi: 10.1038/s41467-019-11105-z (PMC6667477; doi:10.1038/s41467-019-11105-z)
Supplement: Supplementary file 1 — Supplementary Information [file 41467_2019_11105_MOESM1_ESM.pdf]

SUPPLEMENTARY INFORMATION

Stimulation of CRISPR-mediated homology-directed repair by an engineered RAD18 variant

Nambiar et al.

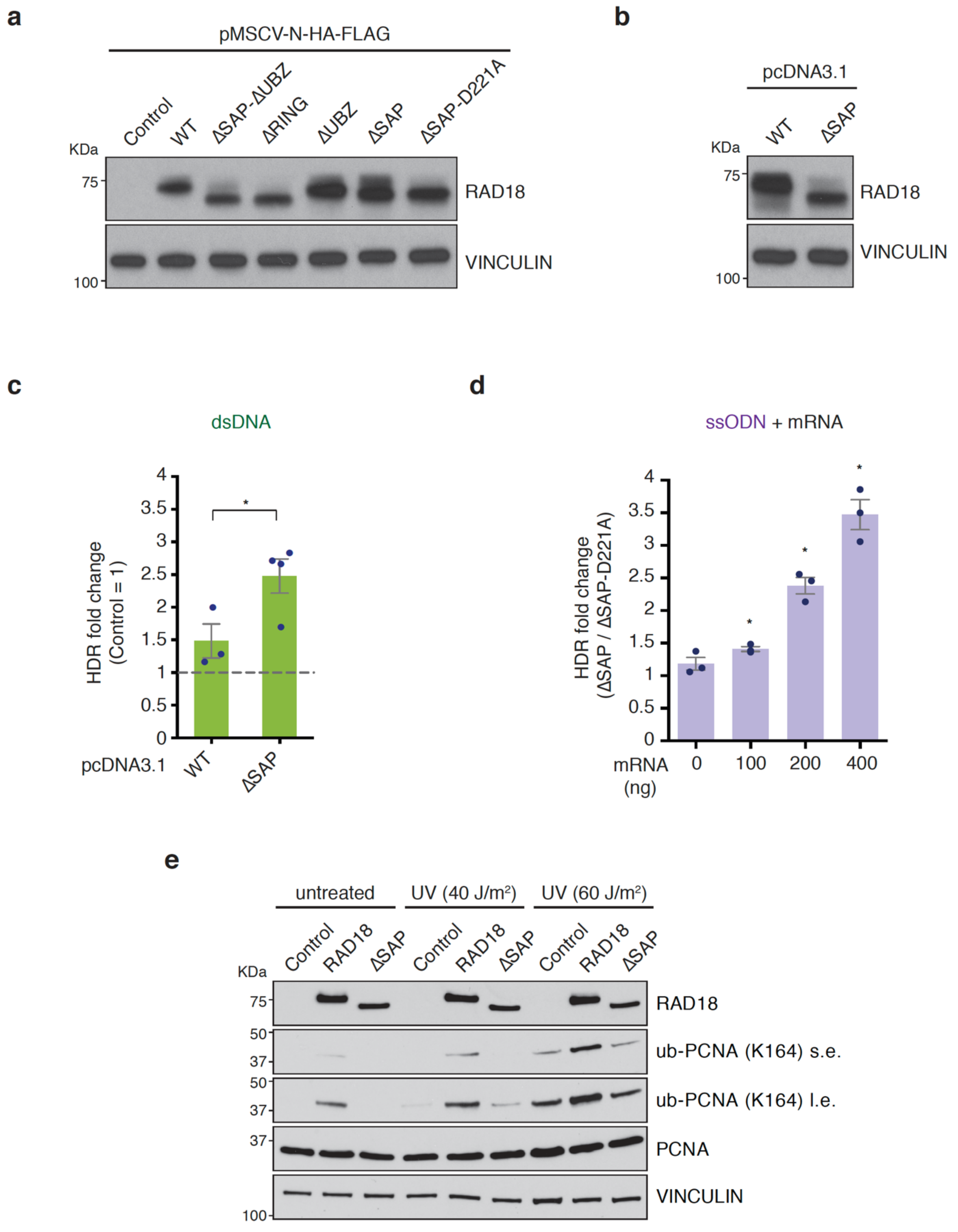

Supplementary Figure 1. HDR stimulation and PCNA ubiquitination induced by WT and mutant RAD18 variants expressed in HEK293T cells. **a** Protein levels of WT and mutant RAD18 upon transfection of RAD18-expressing pMSCV-N-HA-FLAG vectors into HEK293T cells carrying the BFP reporter. Cell lysates were analyzed by western blotting using anti-RAD18 and anti-vinculin

antibodies. The size (KDa) of protein markers is indicated. Transfected cells were subjected to the HDR assays shown in Fig. 2b, c. **b** Protein levels of WT RAD18 and RAD18-ΔSAP upon transfection of RAD18-expressing pcDNA3.1 plasmids into HEK293T cells carrying the BFP reporter. Western blotting was conducted as in **a**. **c** CRISPR-mediated HDR levels measured at the BFP reporter in HEK293T cells transfected with plasmids expressing Cas9 and sgRNA, dsDNA donor, and pcDNA3.1 vectors expressing WT RAD18 or RAD18-ΔSAP, or an empty vector control. HDR frequency was determined by quantifying the percentage of GFP^+ cells, as described in Fig. 1f, g. The values of individual experiments were normalized to the empty vector condition (dashed line) and presented along with the mean ± s.e.m. (n≥3). Statistical significance was calculated using a one-way analysis of variance with Tukey's test, as in Fig. 1f, g (*$p<0.05$). **d** HDR levels measured at the BFP reporter in HEK293T cells nucleofected with Cas9 mRNA, sgRNA and ssODN, along with increasing amounts (0 – 400 ng) of RAD18-ΔSAP or RAD18-ΔSAP-D221A mRNA. HDR frequency was determined by quantifying the percentage of GFP^+ cells, as in **c**. The values of individual experiments were normalized to the ΔSAP-D221A mRNA condition and presented along with the mean ± s.e.m. (n=3). Statistical significance was calculated with a paired t-test comparing the HDR values obtained using RAD18-ΔSAP mRNA versus RAD18-ΔSAP-D221A mRNA (*$p<0.05$). **e** Levels of PCNA and ubiquitinated PCNA in HEK293T cells transfected with vectors expressing WT RAD18, RAD18-ΔSAP or the empty vector control with or without treatment with UV radiation (40 or 60 J/m^2). Western blotting was conducted using anti-PCNA, anti-ubiquitinated PCNA (K164), anti-RAD18 and anti-vinculin antibodies. Two distinct exposures (short exposure, s.e.; long exposure, l.e.) of the anti-ubiquitinated PCNA blot are shown. The size (KDa) of protein markers is indicated.

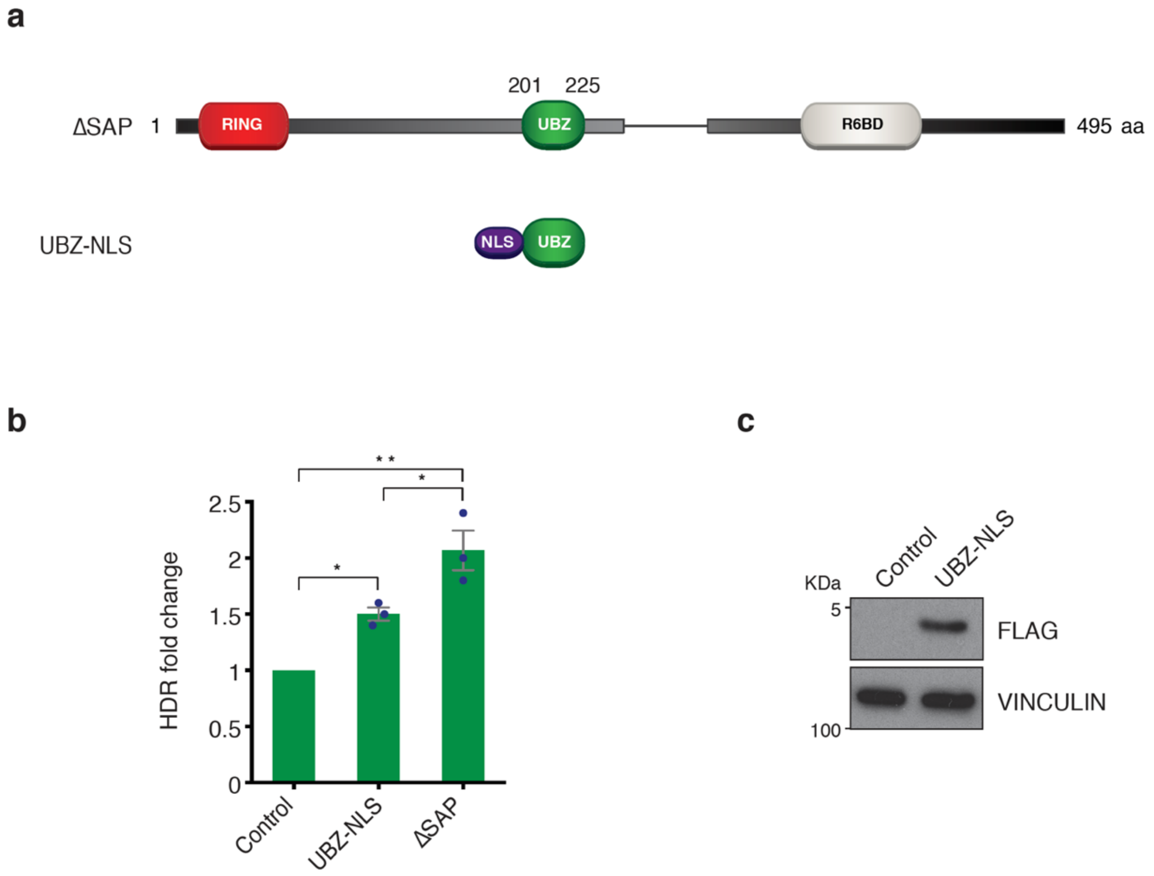

Supplementary Figure 2. CRISPR-mediated HDR levels upon expression of RAD18 UBZ-NLS and RAD18-ΔSAP. **a** Schematic representation of RAD18-ΔSAP and the NLS-tagged UBZ (UBZ-NLS) motif of RAD18. UBZ-NLS has a FLAG tag. **b** CRISPR-mediated HDR levels measured using the BFP reporter stably integrated in HEK293T cells following transfection of dsDNA donor, and vectors expressing Cas9 and sgRNA, along with RAD18-ΔSAP, UBZ-NLS or an empty vector control. 72 h post-transfection, cells were analyzed for GFP fluorescence to determine the frequency of HDR events. The values of individual experiments were normalized to the empty vector condition and presented along with the mean ± s.e.m. (n=3). Statistical analysis was conducted with a one-way analysis of variance with Tukey's multiple comparison test, as in Fig. 1f, g (*$p<0.05$, **$p<0.01$). **c** Expression levels of FLAG-tagged UBZ-NLS in HEK293T cells. Cell lysates were subjected to western blotting using anti-FLAG and anti-vinculin antibodies. The size (KDa) of protein markers is indicated.

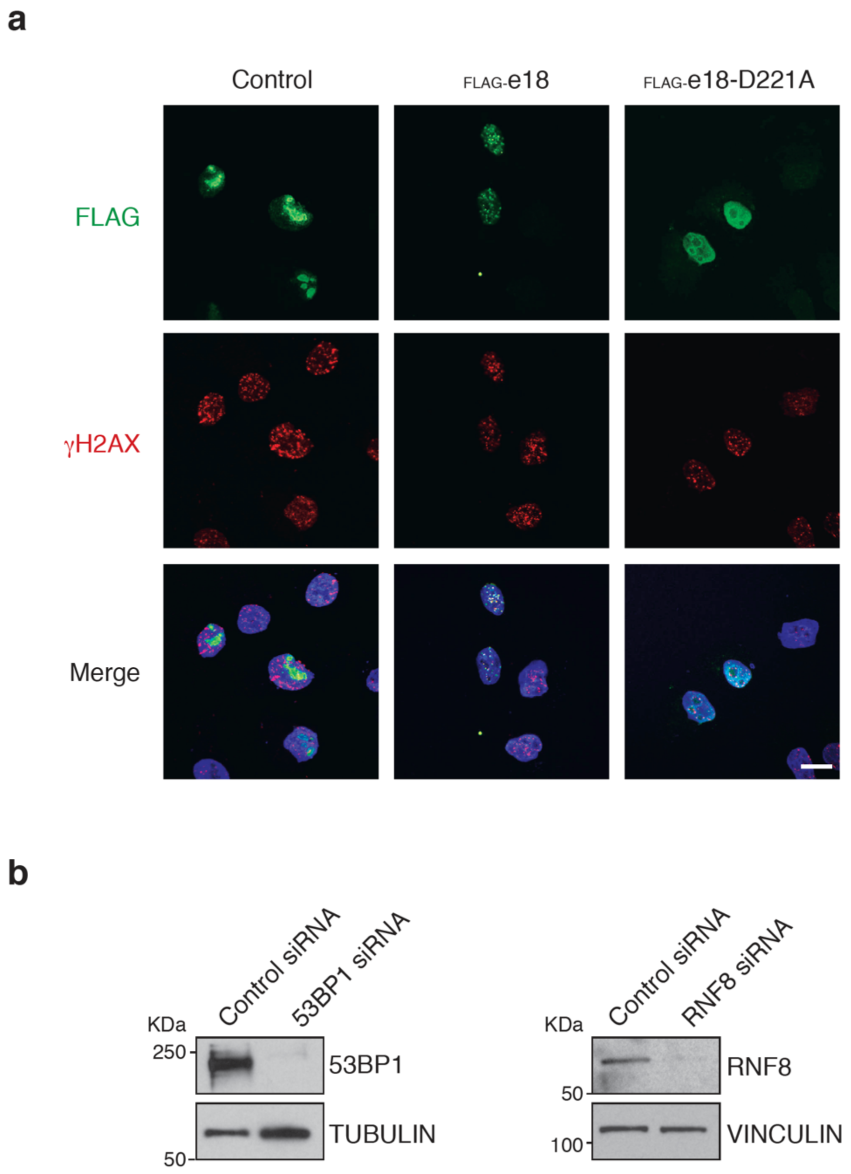

Supplementary Figure 3. γH2AX foci formation and detection of 53BP1 and RNF8 depletion in cells transfected with e18. **a** Representative images showing IR-induced foci of the indicated proteins in U2OS cells transfected with vectors expressing FLAG-tagged e18, e18-D221A mutant or an empty vector control. Cells were irradiated with a 5 Gy dose and fixed 1 h after irradiation. Staining for anti-FLAG (green) and anti-γH2AX (red) and their merge with DAPI staining (blue) is shown. Scale bar: 16 µm. **b** 53BP1 and RNF8 protein levels in HEK293T cells with the BFP reporter upon transfection of 53BP1 or RNF8 siRNA. Cell lysates were analyzed by western blotting using anti-53BP1, anti-RNF8, anti-tubulin and anti-vinculin antibodies. The size (KDa) of protein markers is indicated. Cells depleted of 53BP1 or RNF8 were utilized to conduct the HDR experiments shown in Fig. 3c.

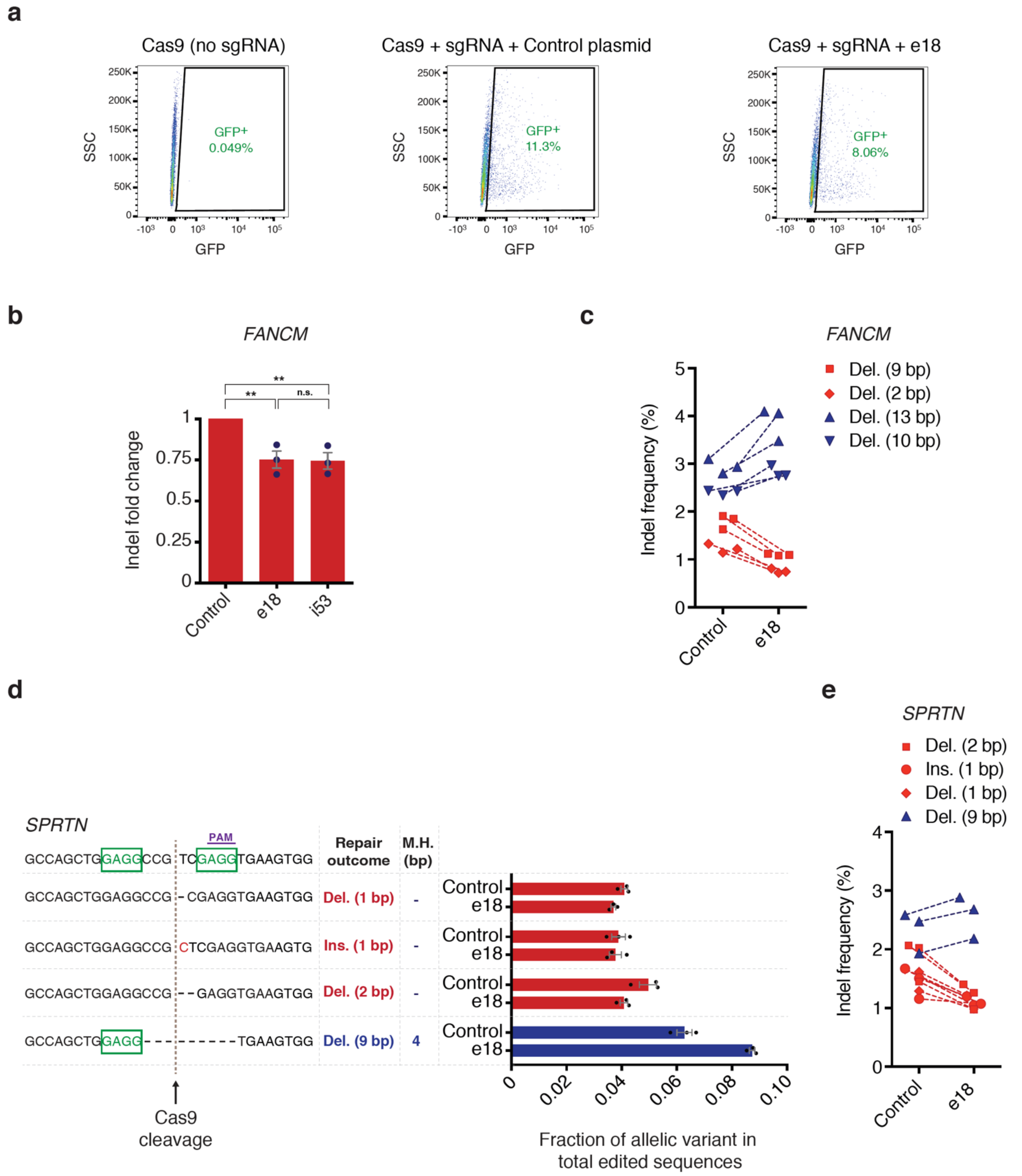

Supplementary Figure 4. Analysis of indel mutation patterns at Cas9-induced DSBs upon e18 expression. **a** Representative flow cytometry plots of end joining experiments with the GFP-2-cut reporter conducted as described in Fig. 4a, b. **b** Indel levels measured by NGS at the *FANCM* locus in HEK293T cells transfected with Cas9/sgRNA-expressing plasmids and ssODN, along with e18, i53

or an empty vector control. The values of individual experiments were normalized to the empty vector control and presented along with the mean ± s.e.m. (n=3). Statistical significance of the data shown was calculated using a one-way analysis of variance with Tukey's multiple comparison test, with a single pooled variance (**$p<0.01$). **c** Frequency of the indicated *FANCM* indel variants shown in Fig. 4e upon treatment with the empty vector control or e18. Individual paired experimental sets are shown. **d** Pattern of indels formed upon treatment of HEK293T cells with Cas9 and sgRNA targeting the *SPRTN* gene, along with either an empty vector control plasmid or e18, as determined by NGS. The wild-type *SPRTN* sequence is shown on the top along with the 4 most frequent mutant alleles resulting from the repair of a Cas9-induced DSB (brown dashed line) in the *SPRTN* locus. Sequences of microhomology (M.H., green boxes) and PAM sequence (purple line) are indicated. The repair outcome (deletion/insertion size) and the length of M.H. utilized for repair are specified. The frequency of the indicated repair products obtained upon transfection of an empty vector control or e18 is represented as fraction of total edited sequences along with the mean ± s.e.m. (n=3). Non M.H.-dependent repair events and M.H.-mediated deletions are depicted in red and blue, respectively. **e** Frequency of the indicated *SPRTN* indel variants upon transfection with the empty vector control or e18. Individual paired experimental sets are shown. The full list of repair products generated at the *SPRTN* targeted site, their frequency and proportion in the edited allele population are compiled in Supplementary Data 2.

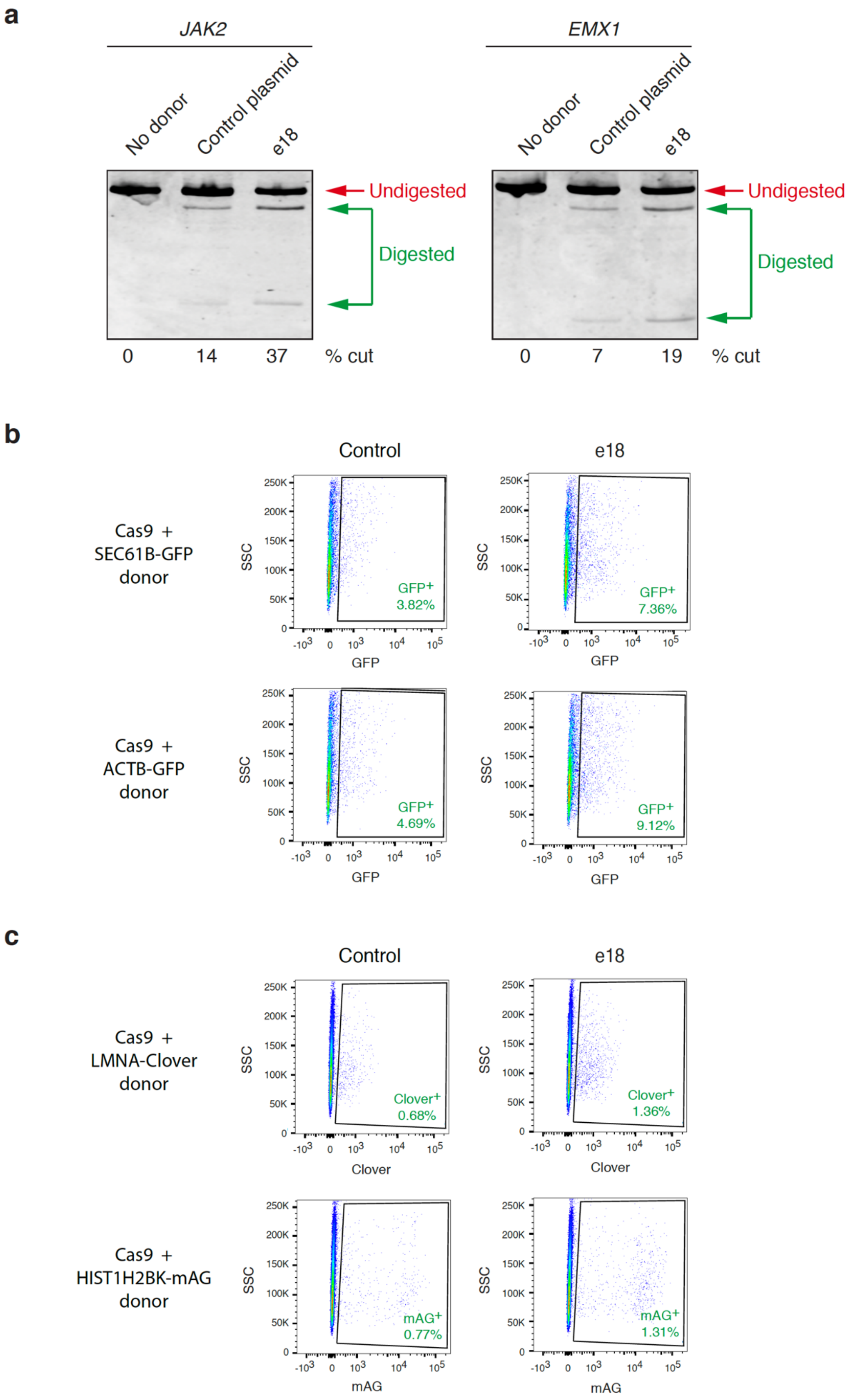

Supplementary Figure 5. RFLP and flow cytometry analysis upon targeting of endogenous loci in cells expressing e18. **a** Representative images of RFLP assays conducted for gene targeting

experiments at the *JAK2* and *EMX1* loci, as described in Fig. 5b, c. Undigested and PmeI-digested DNA products are shown by red and green arrows. **b, c** Representative flow cytometry plots of gene targeting experiments at the *SEC61B*, *ACTB*, *LMNA* and *HIST1H2BK* loci performed as described in Fig. 5d, e.

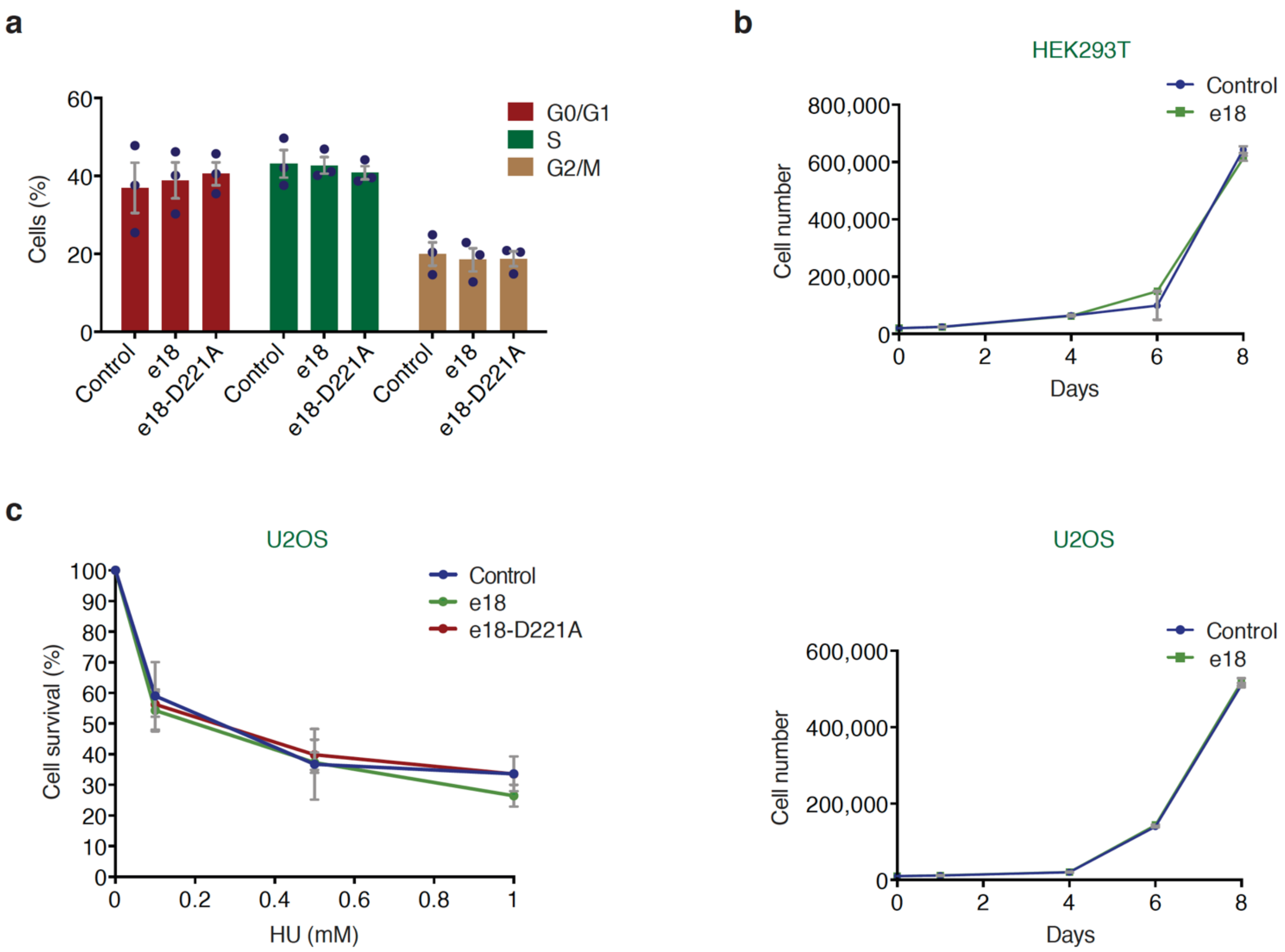

Supplementary Figure 6. Analysis of the effect of e18 expression on cell proliferation. **a** Cell cycle distribution upon e18 expression in HEK293T cells, as measured by propidium iodide staining. HEK293T cells were analyzed by flow cytometry 2 days after transfection with an empty vector control, e18 or the e18-D221A mutant. Results are presented as the mean ± s.e.m. (n=3). **b** Analysis of cell proliferation upon e18 expression. HEK293T (top panel) or U2OS (bottom panel) cells were transfected with an empty vector control or e18 and plated in a 96-well plate. The number of cells per well was quantified at the indicated time points. Pooled data from 3 independent experiments are shown. Error bars represent s.e.m. (n=3). **c** Cell survival analysis on HEK293T cells transfected with the indicated plasmids following treatment with hydroxyurea (HU). The fraction of surviving cells was determined by crystal violet staining after 7 days of HU treatment. Pooled data from 3 independent experiments are shown. Error bars represent s.e.m. (n=3).

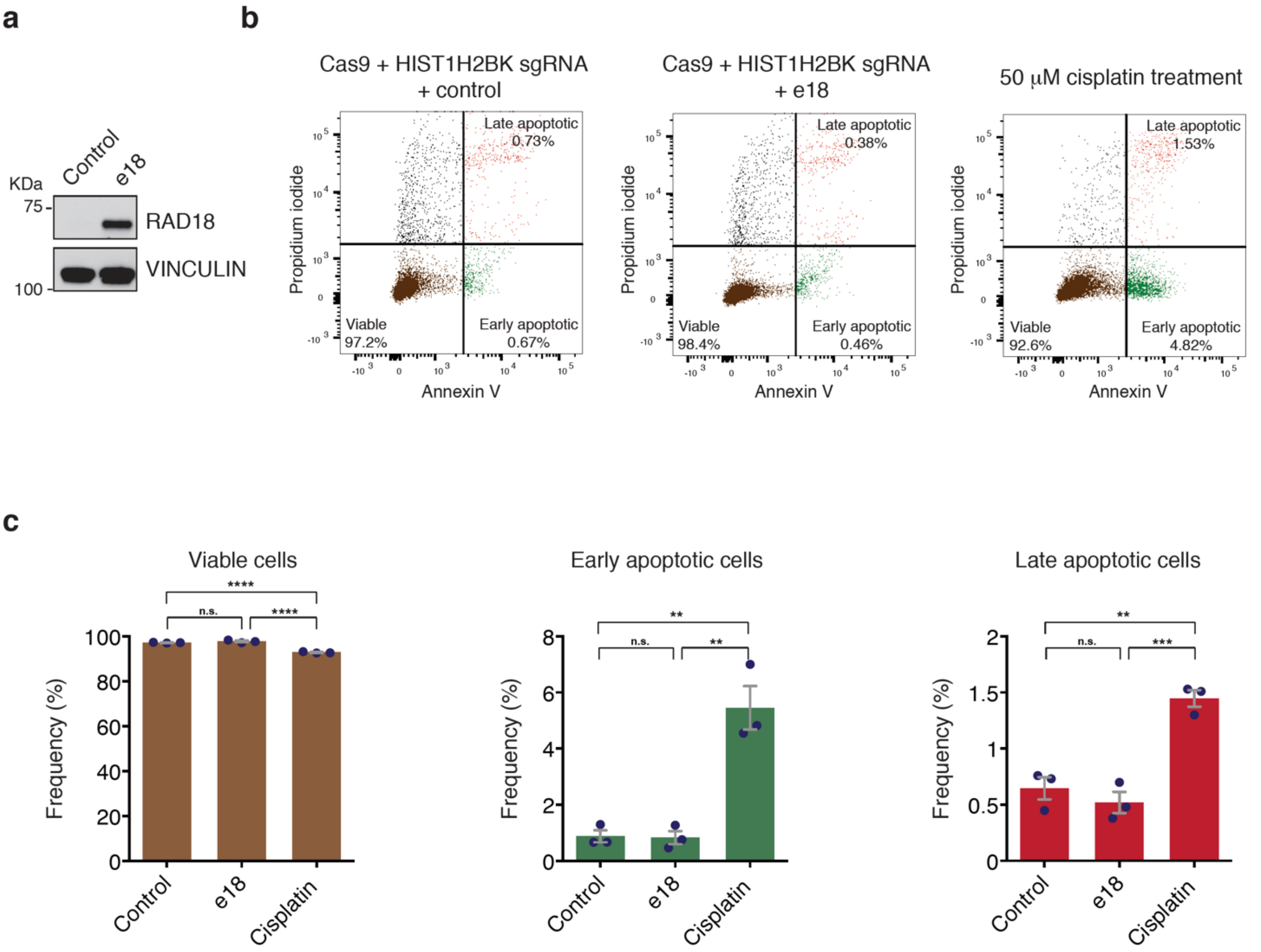

Supplementary Figure 7. Analysis of apoptosis in hESCs expressing e18. **a** e18 expression in diploid hESCs pES12. Cell lysates were analyzed by western blotting using anti-RAD18 and anti-vinculin antibodies. The size (KDa) of protein markers is indicated. **b** Representative flow cytometry plots of apoptosis analysis conducted in pES12 cells 4 days after transfection of Cas9/sgRNA-expressing plasmids, along with either an empty or e18-expressing vector. As a positive control for apoptosis, untransfected cells were treated with cisplatin (50 μM) for 24 h before analysis. Cells were stained with Annexin V and propidium iodide (PI) to evaluate apoptotic cell death by flow cytometry. **c** Percentage of viable (brown), early (green) and late (red) apoptotic pES12 cells under the conditions described in **b,** as determined by Annexin V/PI double staining. Statistical significance was calculated using a one-way analysis of variance with Tukey's multiple comparison test, with a single pooled variance (**p<0.01, ***p<0.001, ****p<0.0001).
