# Supplementary material for: Stimulation of CRISPR-mediated homology-directed repair by an engineered RAD18 variant
Source: Nat Commun. 2019 Jul 30;10:3395. doi: 10.1038/s41467-019-11105-z (PMC6667477; doi:10.1038/s41467-019-11105-z)
Supplement: Supplementary file 6 — Reporting Summary [file 41467_2019_11105_MOESM6_ESM.pdf]

nature research

Corresponding author(s): Alberto Ciccia

Last updated by author(s): Jun 14, 2019

Reporting Summary

Nature Research wishes to improve the reproducibility of the work that we publish. This form provides structure for consistency and transparency in reporting. For further information on Nature Research policies, see Authors & Referees and the Editorial Policy Checklist.

Statistics

For all statistical analyses, confirm that the following items are present in the figure legend, table legend, main text, or Methods section.

n/a	Confirmed	
☐	☒	The exact sample size (n) for each experimental group/condition, given as a discrete number and unit of measurement
☐	☒	A statement on whether measurements were taken from distinct samples or whether the same sample was measured repeatedly
☐	☒	The statistical test(s) used AND whether they are one- or two-sided<br>*Only common tests should be described solely by name; describe more complex techniques in the Methods section.*
☒	☐	A description of all covariates tested
☒	☐	A description of any assumptions or corrections, such as tests of normality and adjustment for multiple comparisons
☐	☒	A full description of the statistical parameters including central tendency (e.g. means) or other basic estimates (e.g. regression coefficient) AND variation (e.g. standard deviation) or associated estimates of uncertainty (e.g. confidence intervals)
☒	☐	For null hypothesis testing, the test statistic (e.g. F, t, r) with confidence intervals, effect sizes, degrees of freedom and P value noted<br>*Give P values as exact values whenever suitable.*
☒	☐	For Bayesian analysis, information on the choice of priors and Markov chain Monte Carlo settings
☒	☐	For hierarchical and complex designs, identification of the appropriate level for tests and full reporting of outcomes
☐	☒	Estimates of effect sizes (e.g. Cohen's d, Pearson's r), indicating how they were calculated

Our web collection on statistics for biologists contains articles on many of the points above.

Software and code

Policy information about availability of computer code

Data collection	N/A
Data analysis	GraphPad Prism v6, FlowJo v10, CRISPResso

For manuscripts utilizing custom algorithms or software that are central to the research but not yet described in published literature, software must be made available to editors/reviewers. We strongly encourage code deposition in a community repository (e.g. GitHub). See the Nature Research guidelines for submitting code & software for further information.

Data

Policy information about availability of data

All manuscripts must include a data availability statement. This statement should provide the following information, where applicable:

- Accession codes, unique identifiers, or web links for publicly available datasets
- A list of figures that have associated raw data
- A description of any restrictions on data availability

The datasets generated during and/or analysed during the current study are available from the corresponding author on reasonable request.

Field-specific reporting

Please select the one below that is the best fit for your research. If you are not sure, read the appropriate sections before making your selection.

☒ Life sciences ☐ Behavioural & social sciences ☐ Ecological, evolutionary & environmental sciences

For a reference copy of the document with all sections, see nature.com/documents/nr-reporting-summary-flat.pdf

Life sciences study design

All studies must disclose on these points even when the disclosure is negative.

Sample size	All experiments shown in this manuscript contain at least 3 biological replicates. No power analyses were done a priori.
Data exclusions	No data has been excluded.
Replication	The effect of e18 to stimulate HDR was reproduced independently by 4 individuals, across two laboratories.
Randomization	There was no randomization in this study.
Blinding	There was no data blinding.

Reporting for specific materials, systems and methods

We require information from authors about some types of materials, experimental systems and methods used in many studies. Here, indicate whether each material, system or method listed is relevant to your study. If you are not sure if a list item applies to your research, read the appropriate section before selecting a response.

Materials & experimental systems

n/a	Involved in the study	
☐	☒	Antibodies
☐	☒	Eukaryotic cell lines
☒	☐	Palaeontology
☒	☐	Animals and other organisms
☒	☐	Human research participants
☒	☐	Clinical data

Methods

n/a	Involved in the study	
☒	☐	ChIP-seq
☐	☒	Flow cytometry
☒	☐	MRI-based neuroimaging

Antibodies

Antibodies used	The source of the antibodies is described in the Methods section.
Validation	The antibodies are commercially available and have been validated by our group as well as others.

Eukaryotic cell lines

Policy information about cell lines

Cell line source(s)	All human transformed cell lines were obtained from ATCC or are derivatives of ATCC. The human pES12 cell line was generated from human oocytes.
Authentication	Transformed cell lines were purchased from ATCC. The pES12 cell line was verified by SNP array, karyotyping and STR genotyping.
Mycoplasma contamination	Cell lines were not tested for mycoplasma contamination.
Commonly misidentified lines (See ICLAC register)	Not applicable.

Flow Cytometry

Plots

Confirm that:

☒ The axis labels state the marker and fluorochrome used (e.g. CD4-FITC).

☒ The axis scales are clearly visible. Include numbers along axes only for bottom left plot of group (a 'group' is an analysis of identical markers).

☐ All plots are contour plots with outliers or pseudocolor plots.

☒ A numerical value for number of cells or percentage (with statistics) is provided.

Methodology

Sample preparation	For all BFP reporter assays and gene tagging experiments, cells were collected from tissue culture plates by trypsinization, diluted into a larger volume of DMEM medium with 10% FBS, spun down, resuspended in PBS and then sorted immediately.
Instrument	BD LSRFortessa
Software	BD FACSDiva for data collection and FlowJo v10 for analysis
Cell population abundance	N/A
Gating strategy	For all assays, live cells were first gated from the FSC/SSC plots, with events of very low or very high FSC and/or SSC being excluded. The boundary between GFP-negative and GFP-positive cells was drawn from the plots of control samples where cells hadn't been transfected with Cas9.

☒ Tick this box to confirm that a figure exemplifying the gating strategy is provided in the Supplementary Information.
