# Supplementary material for: Stimulation of CRISPR-mediated homology-directed repair by an engineered RAD18 variant
Source: Nat Commun. 2019 Jul 30;10:3395. doi: 10.1038/s41467-019-11105-z (PMC6667477; doi:10.1038/s41467-019-11105-z)
Supplement: Supplementary file 7 — Description of Additional Supplementary Files [file 41467_2019_11105_MOESM7_ESM.docx]

**Title:** Supplementary Data 1

**Description:** CRISPR-mediated HDR frequency values upon expression of 204 individual DDR ORFs. HDR fold change values for each ORF, relative to empty vector control, are shown for two independent experiments using ssODN or dsDNA donors in HEK293T cells harboring the BFP reporter. Hits from the screen that modulated HDR ≥1.25- or ≤0.75-fold relative to the empty vector control were carried forward for further validation in HEK293T and HeLa cells expressing the BFP reporter. The HDR fold values relative to empty vector control obtained in the validation experiments are listed. The HDR fold change values for all ORFs are shown in Fig. 1d, e.

**Title:** Supplementary Data 2

**Description:** Indel mutation patterns at endogenous loci targeted by Cas9 in e18-expressing cells. The frequency of allelic variants of 2 endogenous loci (*FANCM* and *SPRTN*) targeted with Cas9/sgRNA is shown for three independent experiments conducted in HEK293T cells transfected with e18 or empty vector control. The data file lists the sequences of the *FANCM* and *SPRTN* variants, the raw frequency of each variant, the frequency of each variant in the total edited allele population and the mean fold change of the frequency of each edited variant observed in cells expressing e18 relative to the empty vector control. Edited variants resulting from MMEJ-mediated repair are highlighted in blue. The length of microhomology for the MMEJ-dependent variants is indicated. The frequency values of the most abundant *FANCM* and *SPRTN* variants resulting from NHEJ- or MMEJ-mediated repair are shown in Fig. 4e and Supplementary Fig. 4c-e. The mean fold change for all edited *FANCM* alleles generated by MMEJ or NHEJ is shown in Fig. 4f.

**Title:** Supplementary Data 3

**Description:** HDR frequency values at endogenous loci targeted by Cas9 in e18-expressing cells. This data file contains the raw HDR frequency values measured in the indicated cell lines and loci, following transfection of Cas9 and sgRNA expression vectors, a donor template and a plasmid expressing e18 or e18-D221A, or an empty vector control. The HDR frequency values for empty vector control and e18 are shown in Figs. 5b-e and 6a.

**Title:** Supplementary Data 4

**Description:** Reagents utilized in this study. This data file lists the ORFs, sgRNAs, ssODNs, DNA primers, siRNAs and antibodies utilized in this study.
